# Generative Large Language Models in Mental Health Care Settings: Systematic Review and Meta-Analysis

**DOI:** 10.2196/87730

**Published:** 2026-08-31

**Authors:** Janni Leung, Benjamin Johnson, Kelsey McRae, Stephanie Fong, Paula Cardona Gonzalez, Caitlin McClure-Thomas, Tianze Sun, Naomi Kern, Yuen Ming Wong, Richard Liu, Gary Chung Kai Chan

**Affiliations:** 1 National Centre for Youth Substance Use Research, The University of Queensland, 31 Upland Road, Brisbane, Queensland, 4067, Australia, 61 429894154

**Keywords:** large language models, generative AI, mental health, primary health care, screening, clinical decision support, psychotherapy, digital health, eHealth, PRISMA, Preferred Reporting Items for Systematic Reviews and Meta-Analyses

## Abstract

**Background:**

General-purpose large language models (LLMs) are increasingly being tested in mental health care, where language is central to assessment, diagnosis, risk evaluation, therapeutic interaction, monitoring, and patient education. However, their clinical usefulness, safety, and readiness for implementation remain uncertain. Existing reviews have largely been descriptive or scoping in nature, and broad health care reviews have not examined in detail the distinctive risks and applications of LLMs in mental health care.

**Objective:**

We aim to systematically review empirical evidence on the use of general-purpose LLMs in mental health care; characterize the clinical tasks, study designs, models, outcomes, and methodological quality of the evidence; and synthesize findings across clinically meaningful task domains, including quantitative synthesis where sufficiently comparable studies were available.

**Methods:**

We followed PRISMA (Preferred Reporting Items for Systematic Reviews and Meta-Analyses) and searched PubMed, Embase, ACM Digital Library, IEEE Xplore, and Google Scholar from November 2022 to March 2026. Eligible studies reported quantitative data evaluating general-purpose LLMs for direct mental health care tasks. Methodological quality was assessed using the Mixed Methods Appraisal Tool and certainty of evidence using GRADE (Grading of Recommendations, Assessment, Development, and Evaluation) domains. Findings were narratively synthesized, and random-effects meta-analyses were conducted on studies that tested LLMs on screening and diagnosis by ChatGPT-4 (OpenAI), ChatGPT-3.5 (OpenAI), and GPT-3 (OpenAI) models for mental health outcomes that reported on specificity and sensitivity. Hartung-Knapp adjustments were applied, and prediction intervals (PIs) estimated.

**Results:**

We included 66 studies, comprising 37 vignette or simulation studies, 22 retrospective studies, and 7 prospective studies. Applications included screening and diagnosis (n=29), clinical decision support (n=14), treatment support (n=10), documentation and monitoring (n=6), patient education (n=4), and patient engagement (n=3). In screening and diagnosis, meta-analysis of 8 studies found that GPT-4 had the higher pooled sensitivity (0.83, 95% CI 0.38‐0.97; 95% PI 0.02‐1.00) than GPT-3.5 (0.70, 95% CI 0.13‐0.97; 95% PI 0.00‐1.00) and GPT-3 (0.61, 95% CI 0.33‐0.82; 95% PI 0.10‐0.96). However, GPT-4 specificity was lower at 0.77 (95% CI 0.52‐0.91; 95% PI 0.10‐0.99). Narrative synthesis suggested that LLMs performed most consistently in structured and linguistically explicit tasks. Certainty of evidence was generally low across domains, although documentation and monitoring reached moderate certainty. Major limitations included indirectness from vignette-based designs, uncertain representativeness, inconsistent outcome reporting, and sparse prospective real-world evaluation.

**Conclusions:**

General-purpose LLMs show promise for selected mental health care applications. However, current evidence remains too heterogeneous, indirect, and uncertain to support routine unsupervised use, particularly for diagnosis, risk assessment, crisis response, or therapeutic interaction. Broad accessibility should not be mistaken for clinical readiness. Future studies should move beyond simulations and retrospective evaluations toward prospective, real-world research assessing safety, reliability, equity, acceptability, clinical outcomes, and implementation in diverse mental health care settings.

## Introduction

### Background

Mental health conditions are a major contributor to global disease burden [1]; yet, access to timely and appropriate care remains limited by persistent shortages in the mental health workforce [2-4]. This is particularly concerning in low-resource settings, where demand for mental health support often substantially exceeds available service capacity. As health systems seek scalable ways to expand access, recent advances in generative large language models (LLMs; eg, ChatGPT [5,6]) have attracted growing interest as tools that may assist selected aspects of mental health care delivery.

Mental health care is a particularly important setting for the evaluation of generative LLMs. For mental health care, spoken and written language underpin diagnostic assessment, risk evaluation, case formulation, therapeutic alliance, and monitoring of treatment response [7]. Several key symptoms of mental disorders are themselves expressed through language, including disorganized speech in psychosis and increased talkativeness in mania [8]. As a result, mental health care is especially exposed to both the opportunities and risks of language-based AI systems. This creates clear potential for LLMs to support mental health care, but also important risks. Errors in interpretation, hallucinated content, limited contextual understanding, and unsafe conversational responses may directly affect clinical judgment, patient safety, and therapeutic trust [8,9].

Research on LLMs in mental health has expanded rapidly since the public release of ChatGPT in late 2022. Previous reviews show that these systems have already been explored across a broad range of applications, including early detection and screening from text, conversational agents, psychoeducation, clinician-facing decision support, therapy-related tasks, and documentation-related uses [9-13]. However, these reviews also indicate that the field remains at an early stage and that much of the existing evidence is not yet well suited to informing clinical implementation. Many studies have relied on prompt-based experiments, vignettes, simulated conversations, cross-sectional comparisons, or evaluations of chatbot responses, with fewer studies using real patient care data or prospective designs embedded in clinical settings [9,14,15]. More broadly, reviews of LLMs in health care have similarly found that evaluations have focused heavily on question answering and accuracy, with limited use of real patient care data, substantial heterogeneity in tasks and outcomes, and inconsistent evaluation methods [5,6].

The rapid evolution of LLM technology further strengthens the rationale for reassessing the evidence base. Earlier reviews identified several limitations in the use of LLMs for mental health care, including inconsistent performance across extended interactions due to limited memory, limited clinical relevance of single-turn question-answer evaluations, and broader concerns regarding transparency and explainability [9,14,16]. Since then, newer generations of LLMs have introduced much larger memory capacity [17], stronger multiturn interaction capacity, and reasoning-oriented variants that can produce more explicit stepwise responses (eg, OpenAI’s o1 [18]). As such, some limitations identified in earlier studies may now be less pronounced, but the updated model may pose new risks. Consequently, conclusions based largely on older generations of models may not fully capture the capabilities and risks of newer LLM systems.

### Study Aims

This systematic review aimed to synthesize empirical evidence on the use of general-purpose LLMs in mental health care. Specifically, we aimed to identify the mental health care tasks for which these models have been evaluated; characterize the study designs, data sources, models, prompting approaches, comparators, mental health conditions, and outcome measures used; synthesize findings across clinically meaningful task domains; assess methodological quality and certainty of evidence; and consider implications for clinical practice and future research. Where groups of studies were sufficiently comparable in task, model, outcome domain, and reported performance metrics, we conducted quantitative synthesis. We focused on general-purpose LLMs because they are widely accessible and therefore among the models most likely to be used ad hoc by clinicians, patients, and health systems, making their evaluation especially relevant to implementation, safety, governance, and equity.

This review adds to existing broad reviews of LLMs and generative AI in health care by focusing specifically on direct mental health care applications, where language is both the medium of care and a central source of clinical information [5,19]. It also extends previous mental health-specific reviews, which have generally been descriptive or scoping in nature, by organizing the evidence into clinically meaningful task domains, distinguishing vignette-based, retrospective, and prospective evaluations, assessing certainty of evidence, and conducting quantitative synthesis only where sufficiently comparable evidence was available [9]. This approach clarifies not only where general-purpose LLMs appear promising, but also where the evidence remains too heterogeneous, indirect, or uncertain to support routine clinical implementation.

## Methods

### Search Strategy

This systematic review and meta-analysis was conducted in accordance with the PRISMA (Preferred Reporting Items for Systematic Reviews and Meta-Analyses) guidelines (see Checklist 1 for PRISMA checklist; see Checklist 2 for PRISMA-S [Preferred Reporting Items for Systematic Reviews and Meta-Analyses literature search extension] checklist) [20]. The review protocol was registered on PROSPERO (International Prospective Register of Systematic Reviews) [CRD420251056593], which was a broader review on AI applications in any primary health care setting. This current paper deviates from the broader PROSPERO in that we focus on clinical applications in mental health care settings. This narrower focus was adopted for the current review because of the rapid expansion of original studies in mental health and the need for a dedicated synthesis of this emerging evidence base.

We searched PubMed, Embase, ACM Digital Library, IEEE Xplore, and Google Scholar for eligible studies. We also manually searched the reference lists of relevant systematic reviews and included papers to identify additional records. The search combined terms related to LLMs (eg, “large language model,” “ChatGPT,” “GPT,” “Claude,” and “Gemini”) with terms related to mental health (eg, “mental health,” “mental disorder,” “depression,” “anxiety,” “suicide,” “psychiatry,” and “psychotherapy”). Full search strings are in Section S2 (Multimedia Appendix 1). For Google Scholar, results were screened in order of relevance until no further potentially eligible studies were identified.

The search was first conducted on January 11, 2025, and updated on June 26, 2025, and again on March 16, 2026. No language restrictions were applied at the search stage or in exclusion criteria, but all included studies were published in English.

### Selection Criteria

The selection criteria were developed based on population or setting, intervention or exposure, outcomes, and study types (Section S1, Multimedia Appendix 1). The population and setting criteria included studies conducted in primary mental health care settings or scenarios (eg, general practice, family medicine, community health centers, and integrated care) for use by individual or group users, such as patients, primary care providers, and mental health practitioners. We excluded studies on populations or settings not relevant to primary mental health care, for example, individual self-help contexts outside clinical care, social media datasets not linked to a defined clinical or decision-making context, health promotion, academic, psychiatry student examinations, and mental health research applications [21-23].

For intervention or exposure, we included general-purpose LLMs, referring to large-scale, pretrained models trained broadly and designed for multipurpose use, without domain-specific training or restriction to clinical mental health applications, without fine-tuning, and without specific use of retrieval augmentation tools (including but not limited to the ChatGPT series, Claude [Anthropic PBC] series, Llama [Meta] series, PaLM/Gemini [Google LLC] series, and Mistral series). We were open to including papers that applied the general-purpose models to any mental health care setting. We excluded studies focusing only on image generation models, domain-specific or fine-tuned models, or small specialized systems developed for specific projects. Chatbot-based studies were eligible if the chatbot functioned primarily as an interface to a general-purpose LLM. Studies were excluded if they evaluated fine-tuned, custom-trained, or otherwise specialized chatbot systems whose performance was not representative of a general-purpose LLM.

We included any outcomes examined, which, based on previous reviews, included accuracy or performance metrics, time efficiency, objectively measured user satisfaction, impact on clinical decision-making, or patient mental health outcomes. Studies without outcomes quantified were excluded.

Study types included were empirical human studies with quantitative data, such as experimental, observational, implementation, evaluation, or mixed-methods studies with measurable outcomes. Opinion pieces, commentaries, reviews, theoretical frameworks, and purely qualitative studies without quantified outcomes were excluded. Preprints, conference papers, and abstracts were included if they met all the criteria, but those with no quantitative data were excluded.

We included studies published from November 2022 onward, corresponding to the public release and widespread accessibility of ChatGPT, the first widely accessible and publicly available general-purpose LLM, in any language and publication type (including peer-reviewed papers, preprints, and conference papers).

### Study Selection

All records identified through database and supplementary searches were imported into Covidence for deduplication and screening. Title and abstract screening and full-text screening were conducted independently by two reviewers. Interreviewer agreement across screening decisions was 70.5% (10,957/15,542). Disagreements were resolved through discussion, and a third reviewer was consulted when consensus could not be reached. Screening decisions were guided by the predefined eligibility criteria, and common reasons for disagreement were discussed within the review team to support consistent interpretation of the criteria.

To minimize double counting, we examined studies with overlapping authors, datasets, or study designs and descriptions for possible duplication. Where multiple reports appeared to use the same or partially overlapping samples, input data, or outcomes, duplicate reports were excluded, and the most relevant or complete report was retained. Studies based on the same datasets were retained if different LLMs were tested or if there were different mental health outcomes.

### Data Extraction

A standardized data extraction form was developed and piloted on 3 included studies before formal extraction commenced. Data were extracted by one reviewer and independently checked by a second reviewer. Any discrepancies were resolved through discussion, with consultation from a third reviewer when needed.

The extracted information included study characteristics (eg, country or region, study design, clinical setting or scenario, data source or population, and target mental health condition), intervention characteristics (eg, model name, model type, prompting approach, and primary mental health care task), and evaluation characteristics (eg, comparator or reference standard, outcome measures, and key findings).

### Data Analysis

Methodological quality and risk of bias were appraised using the Mixed Methods Appraisal Tool (MMAT), which was selected because it provides design-specific criteria applicable to different study designs [24]. MMAT ratings were completed by 1 reviewer and independently checked by a second reviewer. Any disagreements were resolved through discussion until consensus was reached.

A narrative synthesis was used to summarize findings across the full set of included studies because substantial heterogeneity was anticipated in study design, clinical context, model type, prompting approach, and outcome measurement. For synthesis, studies were grouped according to the primary mental health care task evaluated based on studies identified, which were screening and diagnosis, clinical decision support, treatment support, documentation and monitoring, patient education, and patient engagement. These groupings were developed post hoc based on the characteristics of the included studies and were used to support structured comparison across task domains. Similarly, we also grouped the studies based on what designs the included papers had used (eg, prospective, retrospective, or vignette-based design). Prospective studies involved real-time data collection or live interaction with LLMs, including both real-world implementation and controlled research interview settings. Retrospective studies analyzed or tested LLMs on preexisting datasets without live deployment. Vignette-based studies evaluated LLMs using constructed scenarios or standardized prompts rather than real-world data.

Whether to conduct quantitative synthesis and meta-analyses was based on criteria used to determine if comparable estimates were available for pooling. This reflected both clinical and statistical considerations. Studies were grouped according to (1) mental health care task type; (2) LLM model tested; (3) mental disorder, symptom domain, or clinical outcome examined, guided where applicable by *DSM* (*Diagnostic and Statistical Manual of Mental Disorders*) or *ICD* (*International Classification of Diseases*) classifications; and (4) the quantitative metrics reported. Meta-analysis was conducted only when at least four estimates were available within the same task group [25].

Based on these criteria, meta-analysis was restricted to studies in the screening and diagnosis task group that reported sensitivity and specificity, or provided sufficient information to derive these estimates. As too few studies were available to conduct disorder-specific meta-analyses, we grouped studies within a broader internalizing distress and suicide-risk outcome family. This grouping included depressive and anxiety disorders, posttraumatic stress disorder (PTSD)–related outcomes, and suicidality-related outcomes. These outcomes were not treated as equivalent diagnoses; rather, they were grouped because they represent clinically overlapping mental health presentations commonly assessed in screening and risk-detection contexts.

As the outcomes to be pooled were sensitivity and specificity estimates, we conducted random-effects bivariate diagnostic test accuracy meta-analyses using the *MIDAS* package in Stata/SE 18 (StataCorp LLC). A bivariate approach was chosen because sensitivity and specificity are correlated and may vary jointly across studies, so modeling both outcomes together in the same model is appropriate. Separate meta-analyses were conducted by LLM models with at least four estimates for pooling, which included GPT-4, GPT-3.5, and GPT-3. Other models did not have sufficiently comparable data for pooling. It should be noted that in this review, we used the term “GPT-version” instead of referring to the generative pretrained transformer (GPT) family model generically as ChatGPT because we considered ChatGPT to be a web interface that allows connection to different versions of the model. We use the term ChatGPT without the version number when the original study authors did not report the version of GPT used. We did not calculate an overall pooled estimate across all models because combining different LLMs into a single summary estimate was not considered meaningful. We planned to conduct sensitivity analyses by excluding outliers, but there were no outliers identified; therefore, the sensitivity analyses were not conducted. From these models, we estimated pooled sensitivity, specificity, positive likelihood ratio, negative likelihood ratio, and diagnostic odds ratio with 95% CIs. Between-study heterogeneity was assessed using Cochran Q and *I*² statistics, and model adequacy was examined using diagnostic plots [26]. We estimated prediction intervals (PIs) and CIs, and Hartung-Knapp adjustments were applied [27,28].

In addition, we used evidence from the MMAT, narrative review, and meta-analyses to determine the current level of evidence for each of the mental health care tasks that LLMs had been tested on, based on the GRADE (Grading of Recommendations, Assessment, Development, and Evaluation) domains [29].

## Results

### Study Selection

**Figure 1.**
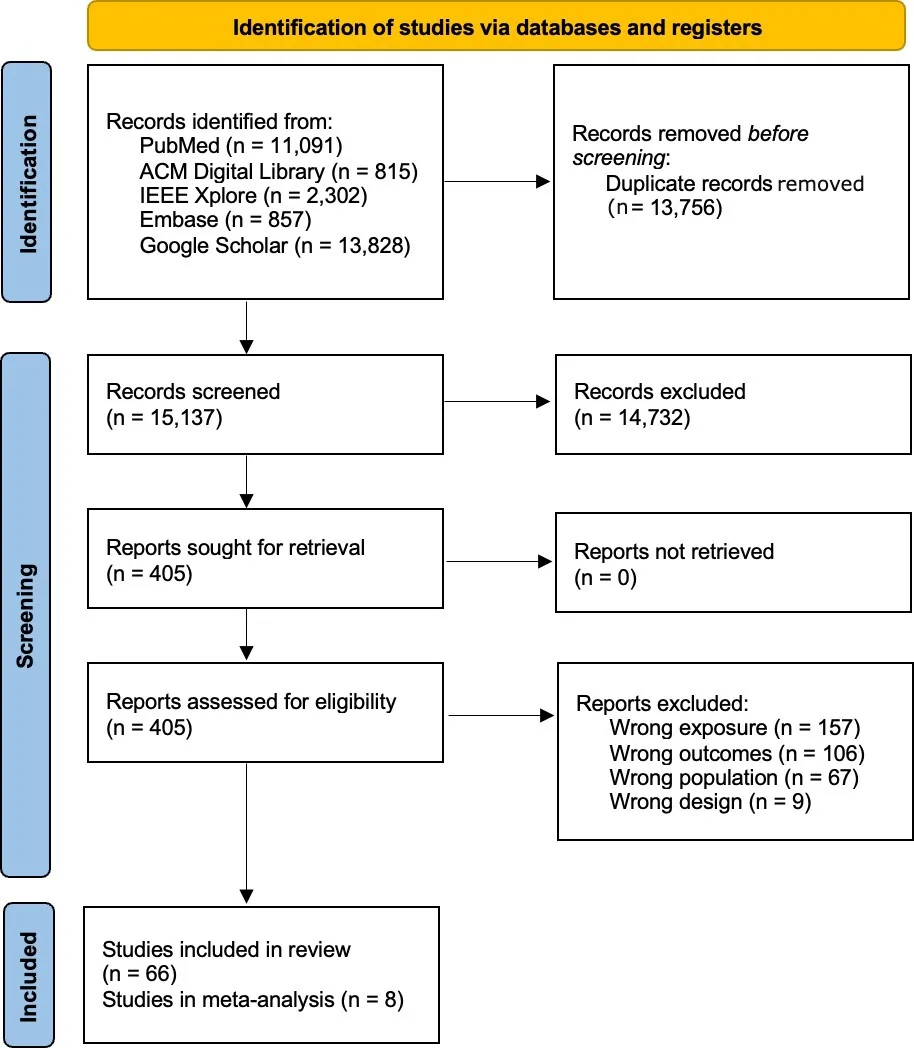
PRISMA (Preferred Reporting Items for Systematic Reviews and Meta-Analyses) flow diagram of study selection.

We identified a total of 28,893 records through database and register searching (PubMed n=11,091; ACM Digital Library n=815; IEEE Xplore n=2302; Embase n=857; Google Scholar n=13,828). After removing 12,777 duplicate records and 979 records for other reasons, 15,137 records were screened, of which 14,732 were excluded at title and abstract screening. We then screened 405 full-texts for eligibility. Finally, a total of 66 studies met the inclusion criteria and were included in the systematic review, with 8 studies included in the meta-analysis (Figure 1).

### Study Characteristics

The characteristics of the 66 included studies are presented in Multimedia Appendix 2. Across the included studies, 48.5% (32/66) of studies were conducted in North America, 15.2% (10/66) of studies in Europe, 19.7% (13/66) of studies in East Asia, 18.2% (12/66) of studies in the Middle East, 4.5% (3/66) of studies in South Asia, and 3% (2/66) of studies in Oceania; some studies spanned multiple regions.

By study design, 56.1% (37/66) of studies were vignette or simulation studies, 33.3% (22/66) of studies were retrospective studies, and 10.6% (7/66) of studies were prospective studies. By application type, 43.9% (29/66) of studies focused on screening and diagnosis, 21.2% (14/66) of studies on clinical decision support, 15.2% (10/66) of studies on treatment support, 9.1% (6/66) of studies on documentation and monitoring, 6.1% (4/66) of studies on patient education, and 4.5% (3/66) of studies on patient engagement.

Target conditions were most commonly general or nonspecific mental health presentations, examined in 31.8% (21/66) of studies, followed by depression in 28.8% (19/66) of studies and suicidality or suicide risk in 16.7% (11/66) of studies. Anxiety-related conditions and schizophrenia or psychosis were each examined in 7.6% (5/66) of studies, bipolar or other mood disorders in 6.1% (4/66) of studies, and PTSD in 4.5% (3/66) of studies. Autism spectrum disorder, obsessive-compulsive disorder (OCD), and psychiatric emergencies or acute psychiatric crises were each examined in 3% (2/66) of studies. Attention-deficit/hyperactivity disorder and substance use or addiction psychiatry were each examined in 1.5% (1/66) of studies. As some studies addressed more than one target condition, these categories were not mutually exclusive.

### Quality Assessment

Based on the MMAT appraisal, methodological quality was generally acceptable, although study quality varied across designs (Section S6 in Multimedia Appendix 1). Among the quantitative nonrandomized studies, 60.4% (29/48) met at least 4 of the 5 MMAT criteria. The most common limitation was sample representativeness: this criterion was rated as unclear in 31.3% (15/48) of studies and not met in 12.5% (6/48) of studies, suggesting potential limitations in generalizability. Control for confounding was reported in 31.3% (15/48) of studies. Among the quantitative descriptive studies, 75% (6/8) of studies met at least 4 of the 5 MMAT criteria, with the main concerns relating to sample representativeness and nonresponse bias. Among the mixed methods studies, 90% (9/10) met at least 4 of the 5 MMAT criteria. Where mixed methods criteria were not fully met, this was usually due to limited justification for the mixed methods design, insufficient integration of qualitative and quantitative findings, or inadequate discussion of divergences between components. Overall, the evidence base was methodologically heterogeneous, and the principal concern was uncertainty regarding representativeness rather than pervasive major flaws in study conduct. The full assessment can be seen in Section S5 (Multimedia Appendix 1). In the following section, studies are summarized by application types.

### Screening and Diagnosis

Screening and diagnosis referred to tasks in which LLMs were used to classify or identify a mental health condition or estimate symptom risk or severity. We identified 29 studies on screening and diagnosis, including 14 retrospective, 13 vignette, and 2 prospective studies.

The 29 studies used heterogeneous methods to evaluate LLM performance in mental health screening and diagnostic classification, including retrospective analyses of patient narrative [30], clinical interview transcripts [31-36], diary entries [37], hospital clinical notes and discharge summaries [38-40], sentence completion narratives [41], inpatient psychiatric admission narratives [42], and structured questionnaire responses [43]. In addition, prospective studies used LLM-derived sentiment ratings from brief written responses [44] and depression screening through conversations [45]. The remaining studies were vignette-based or other simulated evaluations, including *DSM-5* (*Diagnostic and Statistical Manual of Mental Disorders* [Fifth Edition]) or *DSM-5-TR* (*Diagnostic and Statistical Manual of Mental Disorders* [Fifth Edition, Text Revision]) psychiatric diagnostic vignettes [46-48], suicidality and suicide-risk scenarios [48-53], childhood anxiety vignettes [54], and OCD diagnostic vignettes [55,56]. Reference standards and comparators were similarly diverse, including validated symptom scales and thresholds [30-33,35-37,41,43], psychiatrist adjudication or clinical diagnosis [37,45], human annotation with physician validation [38], benchmark *DSM*-based vignette diagnoses [46-48], and mental health professional comparators [51,53,54,57,58].

Across the vignette-based screening and diagnosis studies, LLM performance was mixed. Stronger results were reported in more narrowly defined vignette tasks, such as childhood anxiety disorders, where LLMs often matched or exceeded human comparators or achieved high diagnostic accuracy [54,55,57]. Performance was also strong in OCD studies, where LLMs achieved high diagnostic accuracy and outperformed human comparators [55,56]. In broader psychiatric vignette studies, GPT-3.5 produced broadly acceptable responses across 100 psychiatric case vignettes [59], while newer frontier models showed moderate-to-strong diagnostic performance, with better reasoning associated with greater diagnostic accuracy [60]. Similarly, in a broader psychiatric vignette set, performance was high for depression, social phobia, and PTSD, but weaker for schizophrenia [58].

Structured approaches also improved performance in some cases, such as self-verification prompting, which increased positive predictive value in *DSM-5-TR* case diagnosis in a study [48], but reduced sensitivity [61]. However, important limitations were mentioned in some studies, including weaker performance for disorders in which the diagnosis depends on the symptoms as well as the timing and course of the symptom presentations, such as peripartum depression and acute stress disorder [47]. In addition, limitations mentioned included variability across diagnostic categories and demographic bias [46], delayed escalation and limited crisis referral in simulated suicidality scenarios [49], and systematic deviation from professional norms in suicide-risk judgments, with risks over- or underestimated depending on model type [50,51]. Other vignette-based studies further highlighted variability in referral decisions, intervention stability, and benchmark performance across broader psychiatric or suicide-related tasks [54,62].

Across the retrospective studies, findings were similarly mixed. LLMs performed well for autism spectrum disorder, depression, and PTSD estimation from interviews and inpatient psychiatric diagnosis from admission narratives, although conventional models still outperformed LLMs in some settings [32,34,42,63]. Some studies also reported promising performance for symptom scoring from interview transcripts and suicide-risk estimation from discharge summaries, although discrimination for suicide-risk prediction remained only modest [39,40]. A retrospective study also showed strong performance for binary condition identification from hospital clinical notes, as illustrated by high sensitivity and specificity for depression extraction in 1 small emergency health record study [38]. However, performance was weaker in other applications, including childbirth-related PTSD screening, where sensitivity was low despite high specificity, and adolescent suicidal-ideation estimation, where traditional machine-learning models slightly outperformed LLM-based approaches [30,43]. Other retrospective studies also showed only moderate performance for depression and suicide-risk prediction from sentence-completion narratives and for extraction of mental health causes, although prompting refinements improved results in some settings [33,41,64].

In the 2 prospective studies, a GPT-based fast screen showed high agreement with clinical diagnosis and outperformed the validated Patient Health Questionnaire scale for screening for depression, but in a very small sample [45]. In a separate prospective study, LLM-derived sentiment ratings from brief written responses were associated with both current and future depression severity and significantly predicted short-term worsening, suggesting potential utility for language-based mood monitoring rather than direct diagnosis [44].

For consideration in the meta-analysis, of the 29 studies on screening and diagnosis summarized above, 8 provided sufficiently comparable data on depression and anxiety disorders, PTSD-related outcomes, and suicidality-related outcomes included [30,31,33,37,38,43,45,46]. Reference standards varied across studies and included validated symptom scales [30,31,33,43], symptom scale thresholds with psychiatrist adjudication [37], psychiatrist clinical diagnosis [45], manual human annotation with physician validation [38], and *DSM-5*-based vignette diagnoses [46].

Visual inspection of the meta-analyses’ diagnostic outputs did not suggest concerns about the model fit, and funnel plot asymmetry tests were not statistically significant (GPT-4 *P*=.79, GPT-3.5 *P*=.31, GPT-3 *P*=.42), noting the small number of studies (Table 1; Sections S2-S4, Multimedia Appendix 1). The bivariate meta-analysis found that GPT-4 had the highest pooled sensitivity (0.83, 95% CI 0.38‐0.97; 95% PI 0.02‐1.00), but lower specificity (0.77, 95% CI 0.52‐0.91; 95% PI 0.10‐0.99) than GPT-3.5 and GPT-3, but with large CIs and PIs. GPT-3.5 showed a pooled sensitivity of 0.70 (95% CI 0.13‐0.97; 95% PI 0.00‐1.00) and specificity of 0.96 (95% CI 0.95‐0.96; 95% PI 0.95‐0.96), while GPT-3 showed a pooled sensitivity of 0.61 (95% CI 0.33‐0.82; 95% PI 0.10‐0.96) and specificity of 0.94 (95% CI 0.88‐0.96; 95% PI 0.87‐0.96). GPT-3.5 had the highest positive likelihood ratio (15.90, 95% CI 9.30‐27.30), whereas GPT-4 had the lowest negative likelihood ratio (0.23, 95% CI 0.11‐0.47). Heterogeneity was large. Overall, the meta-analyses showed that GPT-4 had the highest pooled sensitivity, but poor specificity with wide adjusted CIs, and wide PIs indicating substantial between-study variability in expected performance across settings.

**Table 1.** Bivariate meta-analysis of diagnostic test accuracy of LLMs^a^ on internalizing and distress-related mental conditions. There were not enough like-for-like studies for meta-analyses for other LLMs, other areas of mental health care applications, and other mental health outcomes. Forest plots and diagnostic plots are available in Sections S2-S4 in Multimedia Appendix 1.

	GPT-4 (k=4)	GPT-3.5 (k=4)	GPT-3 (k=5)
Pooled sensitivity, pooled estimate (95% CI)	0.83 (0.38-0.97)	0.70 (0.13-0.97)	0.61 (0.33-0.82)
Prediction intervals	0.02-1.00	0.00-1.00	0.10-0.96
Q for sensitivity	35.12^b^	41.44^b^	12.25^c^
I-sq for sensitivity, pooled estimate (95% CI)	91.46 (84.75-98.16)	92.76 (87.34‐98.19)	67.36 (36.28‐98.43)
Specificity, pooled estimate (95% CI)	0.77 (0.52-0.91)	0.96 (0.95‐0.96)	0.94 (0.88‐0.96)
Prediction intervals	0.10-0.99	0.95-0.96	0.87-0.96
Q for specificity	58.10^b^	16.50^b^	6.23^d^
I-sq for specificity, pooled estimate (95% CI)	94.84 (91.33-98.35)	81.82 (64.51‐99.13)	35.80 (0.00‐98.86)
Positive likelihood ratio, pooled estimate (95% CI)	3.60 (2.50-5.30)	15.90 (9.30-27.30)	10.50 (6.20-17.90)
Negative likelihood ratio, pooled estimate (95% CI)	0.23 (0.11-0.47)	0.31 (0.11-0.91)	0.42 (0.25-0.70)
Asymmetry test (*P* value)	.79	.31	.42

a LLM: large language model.

b *P*<.01.

c *P*=.02.

d *P*=.18.

### Clinical Decision Support

Clinical decision support studies included those that tested LLMs in supporting mental health-related clinical judgments, such as prognosis, triage, medication support, or evaluation of therapeutic responses. We identified 14 studies, including 13 vignette-based studies and 1 prospective simulated training study.

The clinical decision support studies covered prognosis, treatment selection, medication support, suicide-response scoring, emergency triage, psychotherapy case-response tasks, counseling benchmarks, and cognitive behavioral therapy (CBT) knowledge and therapist-response tasks. In studies examining schizophrenia and depression vignettes, models generally recommended treatment appropriately, but prognostic judgments varied across models, with GPT-3.5 tending to be more pessimistic than newer models and human comparators [54,65].

Studies of clinical decision support showed promise but also important safety limitations. GPT-4 often selected appropriate antidepressant treatments, but also included contraindicated or poor options in many evaluations [66]. Guideline augmentation improved bipolar treatment selection substantially, although some inappropriate treatments remained [67]. Retrieval-augmented psychiatric medication support also performed well, especially with GPT-4o-based systems [68], and *ICD-11* (*International Classification of Diseases, 11th Revision*) criteria could be translated into highly accurate executable logic after expert correction [55].

Performance was more mixed on complex response-generation tasks. Models showed bias when scoring suicide-intervention responses [69], no model achieved consistently acceptable performance on psychotherapy case-response tasks [70], and LLMs underperformed human reference responses on CBT therapist-response generation despite strong knowledge performance [62]. In psychiatric emergency triage, GPT-4 models showed substantial agreement with clinicians, but some false positives suggested over-triage [71]. Overall, these findings suggest that LLMs have considerable potential for structured mental health clinical decision support, but still require careful oversight for prognosis, safety-sensitive decisions, prescribing, and therapeutically nuanced tasks.

### Treatment Support

Treatment support referred to tasks in which LLMs were used to deliver, simulate, or evaluate therapeutic or supportive interactions, such as psychotherapy-style conversations, CBT-based responses, journaling support, or feedback on helping skills, rather than to screen for a condition or make a formal clinical decision. We identified 10 studies on treatment support, including 5 vignette-based studies, 1 retrospective study, and 4 prospective studies.

The treatment support studies examined a range of applications, including simulated CBT sessions [72], anxiety support through repeated chatbot conversations [73], AI-generated feedback for suicide-prevention role-play training [74], CBT-style cognitive distortion and reframing tasks [75], AI-supported journaling for mental well-being [76], depression treatment recommendations from vignette scenarios [53], psychotherapy knowledge and behavioral activation tasks [58,77], responses to common psychological questions [78], and retrospective CBT-style dialogue generation from psychotherapy transcripts [79]. Reference standards and comparators were similarly varied, including human CBT therapists [72,75], primary care physicians [53], psychotherapists in training [58,77], a licensed clinical psychologist [78], trained human psychology raters [74], human psychotherapy dialogue datasets [79], and user-reported evaluations without a formal comparator [73,76].

Across the 5 vignette-based treatment support studies, findings were generally positive but mixed [50,72,77,78]. LLMs performed moderately to strongly on several structured treatment-support tasks, including CBT-style exercises [75], behavioral activation and psychotherapy case-scenario responses [77], depression treatment recommendation [53], and psychological support question answering [78]. However, important limitations were also identified. Human CBT therapists outperformed GPT-3.5 across multiple CBT competence domains in simulated therapy sessions [72]. In depression vignettes, GPT-3.5 and GPT-4 more often recommended psychotherapy, whereas physicians more often recommended pharmacotherapy [53]. Performance also varied across models, with GPT-4 outperforming GPT-3.5 on common psychological prompts in 1 study [78].

In the 1 retrospective study, LLMs showed feasible CBT-style dialogue generation from psychotherapy transcripts, with stronger performance in multiturn than single-turn settings and further gains when a CBT knowledge base was added [79]. Responses were generally more positive than those of human therapists, suggesting a possible overly positive bias despite reasonable dialogue quality [79].

Across the 4 prospective studies, users and evaluators generally reported favorable experiences with LLM-supported interventions, including anxiety support [73], AI journaling [76], suicide-prevention role-play feedback [74], and psychotherapy training support [58]. Among adults with anxiety disorders, most participants reported that GPT-3.5 understood their anxiety accurately, and ratings of helpfulness, empathy, trustworthiness, and effectiveness were generally favorable, although privacy, ethics, and lack of human connection remained common concerns [73]. AI-supported journaling also received high ratings across counseling skill, behavior, and learning domains [76]. In suicide-prevention role-play training, AI-generated feedback correlated strongly with human ratings, although it tended to score performances more favorably than human raters [74]. In psychotherapy training tasks, LLMs performed similarly to or better than psychotherapists in training on several knowledge and response-quality indicators [53]. Overall, these findings suggest that LLMs may be useful as supportive or training-adjunct tools, but their outputs still require cautious interpretation in sensitive therapeutic contexts [53,73,74].

### Documentation and Monitoring

Documentation and monitoring referred to tasks in which LLMs were used to summarize sessions, generate discharge summaries, standardize notes, extract structured information from free-text records, score written documentation, or monitor symptom change from routine clinical text. We identified 6 studies on documentation and monitoring, including 5 retrospective studies and 1 vignette-based study.

These 6 studies used heterogeneous methods to evaluate LLM performance in documentation and monitoring tasks, including structured summarization of counseling-session transcripts [80], classification of mental health concepts from emergency electronic health record (EHR) text [81], scoring of suicide safety-plan documentation [82], proofreading and structured information extraction from addiction psychiatry notes [83], symptom monitoring from psychiatric EHR language during clozapine treatment [84], and generation of psychiatric discharge summaries from structured case information [85]. Reference standards and comparators were similarly diverse, including human-annotated reference summaries [80], consensus coding by expert clinicians [81], trained clinical coders using a structured scoring algorithm [82], human-annotated gold-standard proofread notes and extraction labels with comparison against non-LLM tools [83], human-rated mental health symptom scores and conventional natural language processing features [84], and human-written discharge summaries by physicians and psychotherapists [85].

Across the 5 retrospective studies, findings were overall positive, with variation by task. LLMs performed well on structured summarization, concept classification, documentation scoring, information extraction, and symptom monitoring from routine clinical language [80-84]. In counseling-session summarization, hallucinations were uncommon across outputs [80]. In emergency EHRs, where clinicians have to file cases under mental health or physical health categories, performance was strongest for the binary mental-vs-physical distinction, with κ=0.77, precision 0.93, recall 0.93, and *F*_1_-score 0.93, but dropped for finer-grained categorization across specific mental and physical health classes [81]. In suicide safety-plan scoring, best *F*_1_-scores ranged from 0.77 to 0.92 across sections [82]. In addiction psychiatry notes, larger LLMs outperformed simpler non-LLM tools for proofreading, and GPT-4o performed strongly on structured information extraction, with a mean *F*_1_-score of 0.97 for substance-class detection, an exact match of 0.96 for time since last use, and mean accuracy of 0.97 for adequacy of time-since-last-use information [83]. In monitoring applications, LLM-derived psychiatric symptom severity scores captured symptom improvement during clozapine treatment and correlated positively with human-rated measures for key psychotic and behavioral features [84]. Overall, performance appeared strongest for more structured extraction and classification tasks, whereas results were mixed for more specific categorization and documentation tasks, suggesting that LLMs performed more consistently in tasks with low complexity.

In the 1 vignette-based study, GPT-4 generated psychiatric discharge summaries from structured case information that were of similar quality to human-written summaries, but did so substantially faster [85]. These findings suggest that LLMs may be used for documentation support to save time, although the evaluation was based on only 2 cases.

### Patient Education

Patient education included tasks in which LLMs were used to answer patient or caregiver questions, provide psychoeducational information, or respond to common mental health information needs and questions. We identified 4 studies on patient education, including 3 vignette-based studies and 1 retrospective study.

These studies examined responses to autism-related consultation questions in a real-world online care setting [86], real-world mental health questions from an online counseling forum evaluated in a simulated format [87], psychosis-related psychoeducation questions [88], and common antidepressant-related questions paired with short patient scenarios [89]. Reference standards included physician responses [86], licensed mental health professional ratings together with comparison against online human therapist responses and LLM-as-judge evaluations [87], psychiatrist and psychologist ratings [88], and blinded psychiatrist ratings comparing LLM and psychiatrist responses [89].

Across the 3 vignette-based studies, GPT-4 provided highly accurate, clear, and clinically useful psychoeducation for psychosis-related questions, although inclusivity was the weakest-rated domain and readability was only moderate [88]. In responses to general mental health questions, a study showed that performance varied across models, with LLaMA-3.3 showing the strongest overall performance, while GPT-4 had lower overall quality and more refusal-style safety disclaimers, and Gemini showed lower empathy despite similar factual consistency [87]. Human therapist responses scored lower overall than LLM responses in that study, although 7%‐14% of LLM outputs still contained unauthorized medical advice [87]. For antidepressant-related questions, GPT-4o performed similarly to psychiatrists in accuracy, was more concise, but produced fewer clear responses, with no significant difference in readability [89].

In the retrospective study, physicians were preferred overall to GPT-4 for autism-related consultation questions, with physicians scoring higher on relevance and usefulness, whereas ChatGPT scored highest on empathy and slightly exceeded physicians on correctness [86]. Overall, these findings on patient education and question answering suggest that LLMs can provide useful patient education responses in mental health settings, but performance remains dependent on the model used and the evaluation domain, with ongoing concerns around clarity, safety, and response quality relative to clinicians [86-89].

### Patient Engagement

Patient engagement referred to tasks in which LLMs were used as interactive, user-facing systems to engage people in mental health conversations or crisis-oriented exchanges, rather than primarily to provide formal diagnosis or structured treatment. Among the 3 patient engagement studies, all 3 were conducted in simulated or vignette-based settings [90-92], and none used retrospective or prospective real-world clinical data.

LLMs were generally perceived as capable of providing supportive or contextually appropriate responses, particularly in lower-risk or general mental health scenarios, but important limitations were identified in more complex or crisis-sensitive situations [69,90,92]. In a psychiatric crisis role-play study, participants rated GPT-3.5 positively overall for helpfulness, pleasantness, and appropriateness, although ratings were lower in psychosis scenarios than in depression or adjustment disorder scenarios [90]. In a study of suicide-related queries, LLMs including Claude 3.5, GPT-4o, and Gemini 1.5 appropriately avoided directly answering very-high-risk questions, but showed limited ability to consistently distinguish between intermediate levels of suicide risk [69]. In a comparison with licensed therapists, LLMs demonstrated some therapeutic elements such as reassurance and psychoeducation, but were judged to be more generic, overly directive, and potentially unsafe in crises [92].

### Certainty of Evidence

Overall, taking into account the quality appraisal, meta-analysis findings, and narrative synthesis, the certainty of evidence was generally low across mental health care tasks (Table 2). The use of LLMs for documentation and monitoring of mental health care had the highest level of evidence, which was moderate. The certainty of evidence was downgraded for other mental health tasks, where evidence was sparse, mixed, or based on simulated designs. A substantial proportion of existing evidence was based on vignette-based studies, limiting the directness and clinical applicability of the evidence. Many other studies were retrospective, which was moderate, but a higher level of evidence would be achieved by having prospective or trial-based studies conducted. Across domains, there were important methodological limitations, including unclear confounding assessment, uncertain sample representativeness, inconsistent outcome measures, unclear publication bias, and imprecision in reported estimates. Heterogeneity was also substantial in the pooled screening and diagnosis studies, with wide CIs and PIs for some outcomes, indicating considerable uncertainty and variation across settings.

**Table 2.** Certainty of evidence based on GRADE^a^ assessments.

Mental health care task	Studies conducted	Summary	Certainty of the evidence
Screening and diagnosis	29 studies; 13 vignette, 14 retrospective, 2 prospective	There was a relatively larger body of evidence available, but findings were mixed across mental health outcomes and task types. Most studies relied on vignette-based or retrospective datasets, with relatively few prospective evaluations. Performance was often promising for some structured classification tasks, but results varied by condition, prompting strategy, and model, and several studies identified important safety or bias-related concerns.	Low
Clinical decision support	14 studies: 13 vignette, 0 retrospective, 1 prospective	Nearly all evidence came from simulated or vignette-based tasks, limiting direct clinical applicability. Findings varied by task and model, and several studies identified clinically important weaknesses, including biased prognostic judgments, over- or undertriage, and inclusion of contraindicated or poor treatment suggestions.	Low
Treatment support	10 studies: 5 vignette, 1 retrospective, 4 prospective	Evidence suggested potential usefulness of LLMs^b^ in supportive or adjunctive treatment roles, but findings varied across contexts, outcomes, and prompting strategies. Prospective studies reported positive experiences with LLMs, although concerns remained regarding privacy, human nuance, and safety in sensitive settings.	Low
Documentation and monitoring	6 studies: 1 vignette, 5 retrospective, 0 prospective	Findings were more consistent in showing positive results, especially for structured extraction, classification, summarization, and documentation tasks. However, all evidence came from retrospective analyses or simulated cases, with no prospective implementation studies.	Moderate
Patient education	4 studies: 3 vignette, 1 retrospective, 0 prospective	Evidence was limited to a small number of mainly simulated studies. Findings generally suggested that LLMs could produce accurate and well-rated psychoeducational or question-answering responses, although safety, clarity, readability, and appropriateness concerns remained. There was a lack of direct real-world evaluation.	Low
Patient engagement	3 studies: 3 vignette, 0 retrospective, 0 prospective	Evidence was sparse and based entirely on simulated interactive scenarios. Findings suggested that LLMs could be engaging and acceptable in some contexts, but concerns remained regarding suitability in complex or crisis-related situations, and the small evidence base limits confidence.	Low

a GRADE: Grading of Recommendations, Assessment, Development, and Evaluation.

b LLM: large language model.

## Discussion

### Principal Findings

This systematic review provides a clinically focused synthesis of empirical evidence on general-purpose LLMs in mental health care. In contrast to broad reviews of LLMs across medicine, this review focuses on a setting in which language is central to clinical assessment, risk evaluation, therapeutic interaction, monitoring, and patient engagement [5,19]. We found that general-purpose LLMs have been evaluated across a wide range of mental health care tasks, including screening and diagnosis, clinical decision support, treatment support, documentation and monitoring, patient education, and patient engagement. The main contribution is a structured assessment of what has been tested, how it has been evaluated, where evidence is beginning to accumulate, and where the evidence remains too heterogeneous or indirect to support clinical implementation. Quantitative synthesis was feasible only for a subset of screening and diagnostic studies with comparable diagnostic test accuracy metrics; all other task domains required narrative synthesis because of substantial variation in tasks, models, data sources, comparators, outcome measures, and reporting. Overall, the findings suggest that general-purpose LLMs show promise for selected mental health care applications, but the evidence remains early, uneven, and insufficient to support routine unsupervised use.

A consistent pattern across the included studies was that general-purpose LLMs performed better in tasks that were relatively structured, constrained, and linguistically explicit [44,80,85,93]. This was most apparent in documentation and monitoring, where models often performed well in summarization, information extraction, concept classification, and other forms of structured text processing [83]. Similar strengths were also evident in some screening, diagnostic, and decision-support tasks when the input format and expected outputs were clear. Many of these tasks, such as classification, extraction, summarization, and question answering, are well-established areas of language model research in which LLMs have shown strong performance across a wide range of domains outside of mental health care [94,95]. The relative strength observed in these more bounded mental health care applications is therefore broadly consistent with the known capabilities of contemporary LLMs as general-purpose language-processing systems [96].

Performance was less reliable in tasks that required nuanced clinical judgment, sustained interpersonal responsiveness, or safe management of ambiguity and risk. In screening and diagnostic applications, results varied across conditions, models, and input formats, and even when overall classification performance appeared encouraging, with better detection rates in more recent LLMs, sensitivity was often less reassuring than specificity, raising concern about missed cases [30,32]. Further, the large PIs indicated that performance cannot be predicted across settings. In treatment support, patient education, patient engagement, and some clinical decision-support applications, LLMs often produced plausible, coherent, and well-structured responses [63,73,76], but this did not always translate into clinically appropriate or trustworthy performance. Reported limitations included generic or overly positive therapeutic responses, inconsistent crisis escalation, weaker handling of temporally nuanced diagnoses, variable prognostic judgments, and concerns about privacy, personalization, and cultural sensitivity [97,98]. Similar concerns have been raised in relation to LLM-supported substance use disorder interventions, where potential benefits such as low-threshold support need to be weighed against risks relating to stigma, demographic bias, hallucinated or unsafe advice, privacy, governance, and the need for robust human oversight [99]. Particularly in patient engagement and crisis-related settings, the evidence remained limited and indirect, being based entirely on simulated scenarios [90-92]. These findings suggest that generating plausible mental health language is not equivalent to demonstrating dependable clinical judgment in more complex or high-stakes settings.

Although this review focused on general-purpose LLMs rather than specialized or fine-tuned mental health models, the broader literature suggests a similar overall pattern. Customized ChatGPT variants and fine-tuned LLMs have shown promise in simulated counseling interactions and in structured tasks such as summarizing counseling notes, discharge summaries, and medication logs [44,73,80,85]. However, these models also continued to raise concerns regarding privacy, ethics, intervention depth, and missed emotional nuance, limiting their reliability as standalone tools [44,80]. Taken together, these findings suggest that both general-purpose and more specialized LLMs share similar limitations in mental health care settings [100].

Our findings are broadly aligned with earlier reviews showing that LLM research in mental health has grown rapidly but has been dominated by early-stage and indirect evaluations [10,12,15]. Prior mental health reviews found that much of the literature consisted of prompt experiments, vignette studies, or evaluations of chatbot responses, with very few prospective studies involving participants [10,12,15]. Broader health care reviews reached similar conclusions, reporting that only a small proportion of studies used real patient care data and that most evaluations relied on examination questions, vignettes, or expert-generated prompts [101]. Further, ethical concerns raised included fairness, bias, being too positive, transparency, and privacy [100]. Compared with these earlier syntheses, the present review suggests an important, although still incomplete, progress in the evidence base. Retrospective clinical data and some prospective real-world evaluations are now beginning to appear in mental health applications of general-purpose LLMs [32,34,42,63]. This review also extends prior work by quantitatively synthesizing a subset of screening and diagnostic studies, whereas many earlier reviews were primarily descriptive, narrative, or scoping in nature [9,13]. Our focus on general-purpose LLMs is also important because these are the most widely accessible models, and therefore the ones most accessible in practice-like settings or used ad hoc by clinicians and patients [102]. This brings practical value to the field by clarifying not only where these models appear most promising, but also where the evidence remains too uncertain, heterogeneous, unpredictable, or indirect to support routine use.

### Limitations

This review should be interpreted in light of several limitations in the available evidence base and synthesis. First, much of the literature was still based on vignette, simulated, or retrospective studies rather than prospective evaluations embedded in routine care, as mentioned above [54,63]. Although these designs are useful for early-stage assessment, they do not fully capture the complexity, uncertainty, and longitudinal nature of real mental health care encounters, in which presentations are more heterogeneous, and interactions unfold over time [10,12,15]. As a result, ecological validity remains limited, and the safety, clinician trust, and real-world uptake of these systems remain insufficiently tested.

Second, the evidence base was highly heterogeneous, with substantial variation in clinical tasks applied, target mental health conditions, LLM model versions, prompting strategies, comparators, data sources, and outcome measures. This limited direct comparison across studies, constrained generalizability, and reduced the extent to which findings could be synthesized quantitatively. These issues also contributed to reduced confidence in the certainty of evidence. There were still only a small number of studies that were based on human data. Future studies will need better causal design, such as well-designed cohort studies with comprehensive confounding adjustment, to estimate the effect of LLMs on various clinical tasks [103,104].

Third, the meta-analysis has important limitations. Quantitative synthesis was possible only after identifying groups of studies that were sufficiently comparable in task, model, outcome domain, and reported performance metric. In practice, this meant that the meta-analysis was restricted to a small number of screening and diagnostic classification studies reporting sensitivity and specificity. The included studies were not sufficiently numerous to support robust subgroup analyses by individual mental disorder, data source, study design, prompting approach, or reference standard. As a result, the pooled estimates should not be interpreted as disorder-specific diagnostic accuracy estimates. The inclusion of related but distinct outcomes, such as depression, anxiety, PTSD-related outcomes, and suicidality-related outcomes, was a pragmatic decision made because disorder-specific pooling was not feasible. Although these outcomes are clinically related and commonly evaluated in screening and risk-detection contexts, they are not interchangeable diagnoses. The wide CIs, wide PIs, and substantial heterogeneity indicate that model performance is likely to vary considerably across settings. The meta-analysis should therefore be viewed as an early and cautious synthesis of the most comparable available evidence, not as definitive evidence of clinical effectiveness or implementation readiness, and also quantifies the lack of data and uncertainty in the evidence.

Fourth, important reporting gaps limited interpretation. Prompting was often incompletely described, even though prompt design can substantially influence model outputs [105]. Without transparent reporting of prompts and prompt refinement, it is difficult to determine whether observed performance differences reflected the model itself, the way it was instructed, or both. Some studies also did not adequately describe recruitment strategies or sampling frames, making it difficult to assess whether included participants or datasets were representative of the populations of interest. In addition, individuals with more complex presentations may have been underrepresented, which may have inflated estimates of model performance [106].

Fifth, most studies were conducted in English, even though LLM performance may differ in non-English settings [107]. This is particularly important because many non-English-speaking settings, including low- and middle-income countries, face greater shortages in mental health care resources and may have the most to gain from scalable digital tools [108]. The limited linguistic and geographic diversity of the evidence therefore constrains the extent to which these findings can be assumed to apply across settings.

Finally, the evidence base is evolving rapidly. Only a limited number of studies evaluated the most recent model variants, including newer GPT-4 and GPT-4o [51,69,71]. Accordingly, the conclusions of this review would need updating as further research becomes available, and AI-assisted living systematic review approaches may help keep pace with the rapid expansion of this literature [109].

### Conclusions

This review provides a clinically focused synthesis of general-purpose LLMs in mental health care. The evidence suggests that these models are most promising for structured, language-based tasks such as documentation, summarization, information extraction, and monitoring, where the input and expected output are relatively constrained. In contrast, evidence remains much less convincing for high-stakes tasks that require nuanced clinical judgment, including diagnosis, risk assessment, prognosis, crisis response, and therapeutic interaction.

The central implication is that broad accessibility should not be mistaken for clinical readiness. General-purpose LLMs may become useful adjunctive tools in mental health care, particularly where they support clinicians rather than replace them. However, current evidence remains too heterogeneous, indirect, and uncertain to justify routine unsupervised use. Future research needs to move beyond vignette-based and retrospective evaluations toward prospective, real-world studies that assess safety, reliability, equity, acceptability, clinical outcomes, and implementation in diverse care settings.

## Supplementary material

10.2196/87730 Multimedia Appendix 1 Review methods, meta-analysis plots, and methodological quality assessment.

10.2196/87730 Multimedia Appendix 2 Summary of studies on the applications of large language models in mental health care (N=66).

10.2196/87730 Checklist 1 PRISMA Checklist.

10.2196/87730 Checklist 2 PRISMA-S Checklist.

## Abbreviations

CBT: cognitive behavioral therapy
*DSM*: *Diagnostic and Statistical Manual of Mental Disorders*
*DSM-5*: *Diagnostic and Statistical Manual of Mental Disorders, Fifth Edition*
*DSM-5-TR*: *Diagnostic and Statistical Manual of Mental Disorders (Fifth Edition, Text Revision)*
EHR: electronic health record
GPT: generative pretrained transformer
GRADE: Grading of Recommendations, Assessment, Development, and Evaluation
*ICD*: *International Classification of Diseases*
*ICD-11*: *International Classification of Diseases, 11th Revision*
LLM: large language model
MMAT: Mixed Methods Appraisal Tool
OCD: obsessive-compulsive disorder
PI: prediction interval
PRISMA: Preferred Reporting Items for Systematic Reviews and Meta-Analyses
PRISMA-S: Preferred Reporting Items for Systematic Reviews and Meta-Analyses literature search extension
PROSPERO: International Prospective Register of Systematic Reviews
PTSD: posttraumatic stress disorder
