# Supplementary material for: Generative Large Language Models in Mental Health Care Settings: Systematic Review and Meta-Analysis
Source: JMIR AI. 2026 Aug 31;5:e87730. doi: 10.2196/87730 (PMC13528826; doi:10.2196/87730)
Supplement: Supplementary file 1 [file ai-v5-e87730-s001.docx]

# Supplementary Material

**Table of Contents**

[Supplementary Material	1](#_Toc230168628)

[Section S1. Inclusion and exclusion criteria and limits	2](#_Toc230168631)

[Section S2. Search strings	3](#_Toc230168632)

[Section S3. GPT-4 meta-analysis plots	5](#_Toc230168633)

[Goodness of fit, bivariate normality, influence analysis, and outlier detection plots	5](#_Toc230168634)

[Forest plot with sensitivity and specificity	6](#_Toc230168635)

[SROC plot	7](#_Toc230168636)

[Funnel plot with regression line	8](#_Toc230168637)

[Likelihood ratio scattergram	9](#_Toc230168638)

[Section S4. GPT-3.5 meta-analysis plots	10](#_Toc230168639)

[Goodness of fit, bivariate normality, influence analysis, and outlier detection plots	10](#_Toc230168640)

[Forest plot with sensitivity and specificity	11](#_Toc230168641)

[SROC plot	12](#_Toc230168642)

[Funnel plot with regression line	13](#_Toc230168643)

[Likelihood ratio scattergram	14](#_Toc230168644)

[Section S5. GPT-3 meta-analysis plots	15](#_Toc230168645)

[Goodness of fit, bivariate normality, influence analysis, and outlier detection plots	15](#_Toc230168646)

[Forest plot with sensitivity and specificity	16](#_Toc230168647)

[SROC plot	17](#_Toc230168648)

[Funnel plot with regression line	18](#_Toc230168649)

[Likelihood ratio scattergram	19](#_Toc230168650)

[Section S6. Quality Assessment using the MMAT 2018	20](#_Toc230168651)

# Section S1. Inclusion and exclusion criteria and limits

| **Category** | **Inclusion criteria** | **Exclusion criteria** |
| --- | --- | --- |
| Population/setting | Primary mental health care settings (e.g., general practice, family medicine, community health centres, integrated care) or studies simulating/recreating primary care-relevant workflows and decision-making tasks. | Studies on psychiatric populations without relevance to primary care tasks, individual self-help outside of mental health care settings, social media datasets unlinked to a defined clinical or decision-making context. |
| Intervention/exposure | General-purpose large language models (GPT series, Claude series, Llama series, PaLM/Gemini series, Mistral series) used for mental health screening, diagnosis, clinical decision support, monitoring, therapeutic adjuncts, or patient education. Multimodal models that utilize large language models as part of their architecture. | Image generation models only, medical-specific language models, small/specialized models developed or trained for specific projects. |
| Study types | Human studies with empirical data, including experimental studies, observational studies, implementation evaluations, user studies/evaluations, quality improvement projects with data, self-reported studies with empirical data, and mixed-methods studies with quantitative components. | Opinion pieces, commentaries, reviews, theoretical frameworks, studies without empirical data, pure qualitative studies without measurable outcomes, conference posters or presentations without abstracts or papers. |
| Outcomes | Measurable outcomes including accuracy/performance metrics, time efficiency, user satisfaction with objective measurements, impact on clinical decisions for mental health care, patient mental health outcomes. | No outcomes measured or no objective measurements used for outcomes. |
| Limits | Published from November 2022 onwards (coinciding with the public release of ChatGPT and widespread accessibility of large language models), in any language, including peer-reviewed publications, preprints, and conference papers/abstracts. | Research/academic applications, medical education applications, purely theoretical or conceptual applications, system development without implementation data. |

# Section S2. Search strings

**Pubmed**

(( Natural Language Processing [MeSH Terms] OR Large language model [Title/Abstract] OR Large language models [Title/Abstract] OR LLM [Title/Abstract] OR LLMs [Title/Abstract] OR generative AI [Title/Abstract] OR Generative Artificial Intelligence [Title/Abstract] OR GenAI [Title/Abstract] OR ChatGPT [Title/Abstract] OR GPT [Title/Abstract] OR Large Language Model Meta AI [Title/Abstract] OR LLaMA [Title/Abstract] ) OR (( Claude [Title/Abstract] OR Bard [Title/Abstract] OR Gemini [Title/Abstract] OR PaLM [Title/Abstract] OR Mistral [Title/Abstract] ) AND ( language [Title/Abstract] OR Google [Title/Abstract] OR intelligence [Title/Abstract] OR AI [Title/Abstract] ))) AND ( Mental Disorders [MeSH Terms] OR Mental Health [MeSH Terms] OR ADHD [Title/Abstract] OR attention-deficit/hyperactivity disorder [Title/Abstract] OR anorexia [Title/Abstract] OR anxiety [Title/Abstract] OR autism [Title/Abstract] OR bipolar [Title/Abstract] OR bulimia [Title/Abstract] OR bulimic [Title/Abstract] OR depress [Title/Abstract] OR depressive [Title/Abstract] OR depression [Title/Abstract] OR eating disorder [Title/Abstract] OR mental disorder [Title/Abstract] OR mental health [Title/Abstract] OR mental illness [Title/Abstract] OR mood disorder [Title/Abstract] OR OCD [Title/Abstract] OR obsessive-compulsive disorder [Title/Abstract] OR panic disorder [Title/Abstract] OR Personality disorder [Title/Abstract] OR psychiatric [Title/Abstract] OR psychiatry [Title/Abstract] OR psychology [Title/Abstract] OR psychological [Title/Abstract] OR psychosis [Title/Abstract] OR psychotic [Title/Abstract] OR PTSD [Title/Abstract] OR Post-traumatic stress [Title/Abstract] OR schizophrenia [Title/Abstract] OR schizophrenic [Title/Abstract] OR suicidal [Title/Abstract] OR suicide [Title/Abstract] OR substance use disorder [Title/Abstract] OR alcohol use disorder [Title/Abstract] OR stimulant use disorder [Title/Abstract] OR alcohol dependence [Title/Abstract] OR substance dependence [Title/Abstract] OR conduct disorder [Title/Abstract] ))

**ACM**

( title:("Large language model" OR "Large language models" OR LLM OR LLMs OR "generative AI" OR "Generative Artificial Intelligence" OR GenAI OR ChatGPT OR GPT OR "Large Language Model Meta AI" OR LLaMA) OR abstract:("Large language model" OR "Large language models" OR LLM OR LLMs OR "generative AI" OR "Generative Artificial Intelligence" OR GenAI OR ChatGPT OR GPT OR "Large Language Model Meta AI" OR LLaMA) OR (	(title:(Claude OR Bard OR Gemini OR PaLM OR Mistral) OR abstract:(Claude OR Bard OR Gemini OR PaLM OR Mistral))	AND	(title:(language OR Google OR intelligence OR AI) OR abstract:(language OR Google OR intelligence OR AI)) ) OR title:("natural language processing" OR "computational linguistics" OR "language models" OR "machine learning") OR abstract:("natural language processing" OR "computational linguistics" OR "language models" OR "machine learning")) AND ( title:(ADHD OR "attention-deficit/hyperactivity disorder" OR anorexia OR anxiety OR autism OR bipolar OR bulimia OR bulimic OR depress OR depressive OR depression OR "eating disorder" OR "mental disorder" OR "mental health" OR "mental illness" OR "mood disorder" OR OCD OR "obsessive-compulsive disorder" OR "panic disorder" OR "Personality disorder" OR psychiatric OR psychiatry OR psychology OR psychological OR psychosis OR psychotic OR PTSD OR "Post-traumatic stress" OR schizophrenia OR schizophrenic OR suicidal OR suicide OR "substance use disorder" OR "alcohol use disorder" OR "stimulant use disorder" OR "alcohol dependence" OR "substance dependence" OR "conduct disorder")

OR abstract:(ADHD OR "attention-deficit/hyperactivity disorder" OR anorexia OR anxiety OR autism OR bipolar OR bulimia OR bulimic OR depress OR depressive OR depression OR "eating disorder" OR "mental disorder" OR "mental health" OR "mental illness" OR "mood disorder" OR OCD OR "obsessive-compulsive disorder" OR "panic disorder" OR "Personality disorder" OR psychiatric OR psychiatry OR psychology OR psychological OR psychosis OR psychotic OR PTSD OR "Post-traumatic stress" OR schizophrenia OR schizophrenic OR suicidal OR suicide OR "substance use disorder" OR "alcohol use disorder" OR "stimulant use disorder" OR "alcohol dependence" OR "substance dependence" OR "conduct disorder") OR title:("mental health" OR psychiatry OR psychology) OR abstract:("mental health" OR psychiatry OR psychology))

**IEEE**

( ( ("Mesh_Terms":"Mental Disorders") OR ("Mesh_Terms":"Mental Health") OR ("Publication Title":"ADHD" OR "Abstract":"ADHD") OR ("Publication Title":"attention-deficit/hyperactivity disorder" OR "Abstract":"attention-deficit/hyperactivity disorder") OR ("Publication Title":"anorexia" OR "Abstract":"anorexia") OR ("Publication Title":"anxiety" OR "Abstract":"anxiety") OR ("Publication Title":"autism" OR "Abstract":"autism") OR ("Publication Title":"bipolar" OR "Abstract":"bipolar") OR ("Publication Title":"bulimia" OR "Abstract":"bulimia") OR ("Publication Title":"bulimic" OR "Abstract":"bulimic") OR ("Publication Title":"depress" OR "Abstract":"depress") OR ("Publication Title":"depressive" OR "Abstract":"depressive") OR ("Publication Title":"depression" OR "Abstract":"depression") OR ("Publication Title":"eating disorder" OR "Abstract":"eating disorder") OR ("Publication Title":"mental disorder" OR "Abstract":"mental disorder") OR ("Publication Title":"mental health" OR "Abstract":"mental health") OR ("Publication Title":"mental illness" OR "Abstract":"mental illness") OR ("Publication Title":"mood disorder" OR "Abstract":"mood disorder") OR ("Publication Title":"OCD" OR "Abstract":"OCD") OR ("Publication Title":"obsessive-compulsive disorder" OR "Abstract":"obsessive-compulsive disorder") OR ("Publication Title":"panic disorder" OR "Abstract":"panic disorder") OR ("Publication Title":"Personality disorder" OR "Abstract":"Personality disorder") OR ("Publication Title":"psychiatric" OR "Abstract":"psychiatric") OR ("Publication Title":"psychiatry" OR "Abstract":"psychiatry") OR ("Publication Title":"psychology" OR "Abstract":"psychology") OR ("Publication Title":"psychological" OR "Abstract":"psychological") OR ("Publication Title":"psychosis" OR "Abstract":"psychosis") OR ("Publication Title":"psychotic" OR "Abstract":"psychotic") OR ("Publication Title":"PTSD" OR "Abstract":"PTSD") OR ("Publication Title":"Post-traumatic stress" OR "Abstract":"Post-traumatic stress") OR ("Publication Title":"schizophrenia" OR "Abstract":"schizophrenia") OR ("Publication Title":"schizophrenic" OR "Abstract":"schizophrenic") OR ("Publication Title":"suicidal" OR "Abstract":"suicidal") OR ("Publication Title":"suicide" OR "Abstract":"suicide") OR ("Publication Title":"substance use disorder" OR "Abstract":"substance use disorder") OR ("Publication Title":"alcohol use disorder" OR "Abstract":"alcohol use disorder") OR ("Publication Title":"stimulant use disorder" OR "Abstract":"stimulant use disorder") OR ("Publication Title":"alcohol dependence" OR "Abstract":"alcohol dependence") OR ("Publication Title":"substance dependence" OR "Abstract":"substance dependence") OR ("Publication Title":"conduct disorder" OR "Abstract":"conduct disorder") ) AND ( ("Mesh_Terms":"Natural Language Processing") OR ("Publication Title":"Large language model" OR "Abstract":"Large language model") OR ("Publication Title":"Large language models" OR "Abstract":"Large language models") OR ("Publication Title":"LLM" OR "Abstract":"LLM") OR ("Publication Title":"LLMs" OR "Abstract":"LLMs") OR ("Publication Title":"generative AI" OR "Abstract":"generative AI") OR ("Publication Title":"Generative Artificial Intelligence" OR "Abstract":"Generative Artificial Intelligence") OR ("Publication Title":"GenAI" OR "Abstract":"GenAI") OR ("Publication Title":"ChatGPT" OR "Abstract":"ChatGPT") OR ("Publication Title":"GPT" OR "Abstract":"GPT") OR ("Publication Title":"Large Language Model Meta AI" OR "Abstract":"Large Language Model Meta AI") OR ("Publication Title":"LLaMA" OR "Abstract":"LLaMA") OR ("Publication Title":"Claude" OR "Abstract":"Claude") OR ("Publication Title":"Bard" OR "Abstract":"Bard") OR ("Publication Title":"Gemini" OR "Abstract":"Gemini") OR ("Publication Title":"PaLM" OR "Abstract":"PaLM") OR ("Publication Title":"Mistral" OR "Abstract":"Mistral") ))

**Embase**

('natural language processing'/exp OR 'large language model*':ti,ab OR llm:ti,ab OR llms:ti,ab OR 'generative ai':ti,ab OR 'generative artificial intelligence':ti,ab OR genai:ti,ab OR chatgpt:ti,ab OR gpt:ti,ab OR 'large language model meta ai':ti,ab OR llama:ti,ab OR ((claude:ti,ab OR bard:ti,ab OR gemini:ti,ab OR palm:ti,ab OR mistral:ti,ab) AND (language:ti,ab OR google:ti,ab OR intelligence:ti,ab OR ai:ti,ab))) AND ('mental disease'/exp OR 'mental health'/exp OR 'attention deficit hyperactivity disorder'/exp OR 'anorexia nervosa'/exp OR 'anxiety disorder'/exp OR 'autism spectrum disorder'/exp OR 'bipolar disorder'/exp OR 'bulimia nervosa'/exp OR 'depression'/exp OR 'eating disorder'/exp OR 'mood disorder'/exp OR 'obsessive compulsive disorder'/exp OR 'panic disorder'/exp OR 'personality disorder'/exp OR 'psychosis'/exp OR 'posttraumatic stress disorder'/exp OR 'schizophrenia'/exp OR 'suicide'/exp OR 'suicidal ideation'/exp OR 'substance use disorder'/exp OR 'alcohol use disorder'/exp OR 'stimulant abuse' OR 'alcohol dependence'/exp OR 'substance dependence'/exp OR 'conduct disorder'/exp OR adhd:ti,ab OR 'attention-deficit/hyperactivity disorder':ti,ab OR anorexia:ti,ab OR anxiety:ti,ab OR autism:ti,ab OR bipolar:ti,ab OR bulimia:ti,ab OR bulimic:ti,ab OR depress*:ti,ab OR 'eating disorder':ti,ab OR 'mental disorder':ti,ab OR 'mental health':ti,ab OR 'mental illness':ti,ab OR 'mood disorder':ti,ab OR ocd:ti,ab OR 'obsessive compulsive disorder':ti,ab OR 'panic disorder':ti,ab OR 'personality disorder':ti,ab OR psychiatric:ti,ab OR psychiatry:ti,ab OR psychology:ti,ab OR psychological:ti,ab OR psychosis:ti,ab OR psychotic:ti,ab OR ptsd:ti,ab OR 'post traumatic stress':ti,ab OR schizophrenia:ti,ab OR schizophrenic:ti,ab OR suicidal:ti,ab OR suicide:ti,ab OR 'substance use disorder':ti,ab OR 'alcohol use disorder':ti,ab OR 'stimulant use disorder':ti,ab OR 'alcohol dependence':ti,ab OR 'substance dependence':ti,ab OR 'conduct disorder':ti,ab)

# Section S3. GPT-4 meta-analysis plots

### Goodness of fit, bivariate normality, influence analysis, and outlier detection plots

### Forest plot with sensitivity and specificity

### SROC plot

### Funnel plot with regression line

### Likelihood ratio scattergram

# Section S4. GPT-3.5 meta-analysis plots

### Goodness of fit, bivariate normality, influence analysis, and outlier detection plots

### Forest plot with sensitivity and specificity

### SROC plot

### Funnel plot with regression line

### Likelihood ratio scattergram

# Section S5. GPT-3 meta-analysis plots

### Goodness of fit, bivariate normality, influence analysis, and outlier detection plots

### Forest plot with sensitivity and specificity

### SROC plot

### Funnel plot with regression line

### Likelihood ratio scattergram

# Section S6. Quality Assessment using the MMAT 2018

| **Quantitative non-randomized** | | | | | | | | | | | | | |  |
| --- | --- | --- | --- | --- | --- | --- | --- | --- | --- | --- | --- | --- | --- | --- |
| Author | | | S1 | | | S2 | | 3·1 | | 3·2 | 3·3 | 3·4 | 3·5 |  |
| Barabas et al., 2025 | | | Y | | | Y | | N | | Y | Y | Y | Y |  |
| Cardamone et al., 2025 [80] | | | Y | | | Y | | Y | | Y | Y | Y | Y |  |
| Danner, 2023 [31] | | | Y | | | Y | | C | | Y | Y | N | Y |  |
| Dergaa et al., 2024 | | | Y | | | Y | | N | | Y | Y | N | Y |  |
| Elyoseph & Levkovich, 2024 [65] | | | Y | | | Y | | C | | Y | Y | N | Y |  |
| Elyoseph et al., 2024 [98] | | | Y | | | Y | | C | | Y | Y | N | Y |  |
| D'Souza et al., 2023 [59] | | | Y | | | Y | | C | | Y | Y | N | Y |  |
| Donnelly et al., 2025 [82] | | | Y | | | Y | | Y | | Y | y | C | Y |  |
| Gargari et al., 2024 [47] | | | Y | | | Y | | Y | | Y | Y | N | Y |  |
| Gireesh et al., 2025 | | | Y | | | Y | | Y | | Y | Y | N | Y |  |
| He et al., 2024 [86] | | | Y | | | Y | | Y | | Y | Y | Y | Y |  |
| Heinz et al., 2023 | | | Y | | | Y | | C | | Y | Y | Y | Y |  |
| Hodson & Williamson, 2024 [75] | | | Y | | | Y | | C | | Y | Y | N | Y |  |
| Hu et al, 2024 [32] | | | Y | | | Y | | C | | Y | Y | N | Y |  |
| Hur et al., 2024 [44] | | | Y | | | Y | | C | | Y | Y | C | Y |  |
| Jain et al., 2024 [76] | | | Y | | | Y | | C | | Y | Y | C | Y |  |
| Jin, 2024 | | | Y | | | Y | | Y | | Y | Y | C | Y |  |
| Janota & Janota, 2024 | | | Y | | | Y | | C | | Y | Y | C | Y |  |
| Kaliosis et al., 2026 | | | Y | | | Y | | Y | | Y | Y | N | Y |  |
| Kim and Wang, 2026 | | | Y | | | Y | | N | | Y | Y | N | Y |  |
| Lauderdale et al., 2025 [51] | | | Y | | | Y | | N | | Y | Y | C | Y |  |
| Lee et al., 2026 [36] | | | Y | | | Y | | Y | | C | Y | C | Y |  |
| Levkovich et al.,2024 [54] | | | Y | | | Y | | Y | | Y | Y | Y | Y |  |
| Levkovich, 2024 | | | Y | | | Y | | Y | | Y | Y | Y | Y |  |
| Lho et al., 2025 | | | Y | | | Y | | Y | | Y | Y | N | Y |  |
| Liu et al., 2025 [64] | | | Y | | | Y | | N | | Y | Y | C | Y |  |
| Lorenzoni et al., 2024 [33] | | | Y | | | Y | | Y | | Y | Y | C | Y |  |
| Marengo and Longobardi, 2025 [43] | | | Y | | | Y | | Y | | Y | Y | C | Y |  |
| Matsumura et al., 2026 [84] | | | Y | | | Y | | Y | | Y | Y | Y | Y |  |
| McBain et al., 2025A [69] | | | Y | | | Y | | Y | | Y | Y | Y | Y |  |
| McBain et al., 2025B [91] | | | Y | | | Y | | Y | | Y | Y | Y | Y |  |
| McCoy and Perlis, 2025A [39] | | | Y | | | Y | | Y | | Y | Y | Y | Y |  |
| McCoy and Perlis, 2025B [40] | | | Y | | | Y | | Y | | Y | Y | C | Y |  |
| Moëll, 2024 | | | Y | | | Y | | C | | Y | Y | Y | Y |  |
| Napitwotzki et al., 2025 | | | Y | | | Y | | Y | | Y | Y | Y | Y |  |
| Nyugen et al., 2025 | | | Y | | | Y | | N | | Y | Y | N | Y |  |
| Perlis et al., 2023 | | | Y | | | Y | | C | | Y | Y | C | Y |  |
| Perlis et al., 2024 [67] | | | Y | | | Y | | C | | Y | Y | C | Y |  |
| Sarma et al., 2025A [48] | | | Y | | | Y | | Y | | Y | Y | C | Y |  |
| Sarma et al., 2025B | | | Y | | | Y | | Y | | Y | Y | Y | Y |  |
| Senturk and Koparal, 2026 [89] | | | Y | | | Y | | C | | Y | Y | C | Y |  |
| Shen et al., 2024 [79] | | | Y | | | Y | | Y | | Y | Y | Y | Y |  |
| Silva and Gomes, 2025 [68] | | | Y | | | Y | | Y | | Y | Y | C | Y |  |
| Shin et al., 2024 [37] | | | Y | | | Y | | Y | | Y | Y | N | Y |  |
| Sun et al., 2026 [42] | | | Y | | | Y | | Y | | Y | Y | C | Y |  |
| Teferra et al., 2025 [35] | | | Y | | | Y | | Y | | Y | Y | C | Y |  |
| Thotapalli et al., 2025 [71] | | | Y | | | Y | | Y | | Y | Y | Y | Y |  |
| Yilanli et al., 2024 [88] | | | Y | | | Y | | C | | Y | Y | C | Y |  |
| **Quantitative descriptive** | | | | | | | | | | | | | |  |
| Author | | | S1 | | | S2 | | 4·1 | | 4·2 | 4·3 | 4·4 | 4·5 |  |
| Alanzi et al., 2024 | | | Y | | | Y | | Y | | C | Y | Y | Y |  |
| Bartal et al., 2024 [30] | | | Y | | | Y | | Y | | C | Y | C | Y |  |
| Bhagat et al., 2024 [38] | | | Y | | | Y | | Y | | Y | Y | Y | Y |  |
| Heston, 2023 [49] | | | Y | | | Y | | Y | | Y | Y | Y | Y |  |
| Kim et al., 2025 | | | Y | | | Y | | Y | | C | Y | Y | Y |  |
| Levkovich & Elyoseph, 2023A [50] | | | Y | | | Y | | Y | | C | Y | Y | Y |  |
| Levkovich & Elyoseph, 2023B [53] | | | Y | | | Y | | Y | | C | Y | C | Y |  |
| Levkovich, 2025 [56] | | | Y | | | Y | | Y | | Y | Y | Y | Y |  |
| **Mixed Methods** | | | | | | | | | | | | | |  |
| **Author** | | | S1 | | | S2 | | 5·1 | | 5·2 | 5·3 | 5·4 | 5·5 |  |
| Acevedo et al., 2026 [72] | | | Y | | | Y | | Y | | Y | Y | Y | Y |  |
| Adhikary et al., 2024 [80] | | | Y | | | Y | | Y | | Y | Y | Y | Y |  |
| Haber et al., 2025 [74] | | | Y | | | Y | | Y | | Y | Y | Y | Y |  |
| Jin et al., 2026 [60] | | | Y | | | Y | | Y | | Y | Y | Y | Y |  |
| Levkovich et al., 2025 [58] | | | Y | | | Y | | Y | | Y | Y | C | Y |  |
| Li et al., 2026 [87] | | | Y | | | Y | | N | | Y | Y | Y | Y |  |
| Scholich et al., 2025 [92] | | | Y | | | Y | | Y | | Y | Y | C | Y |  |
| Tan et al., 2025 [70] | | | Y | | | Y | | N | | Y | Y | Y | Y |  |
| Zeng et al., 2025 [52] | | | Y | | | Y | | N | | N | Y | Y | Y |  |
| Zhang et al., 2025 [17] | | | Y | | | Y | | Y | | Y | Y | C | Y |  |
|  |  |  | |  |  | |  |  |  | | | | |  |

Y Yes, N No, C Can’t determine, NA not applicable

S1: Are there clear research questions? S2: Do the collected data allow to address the research questions? 1·1: Is the qualitative approach appropriate to answer the research question? 1·2: Are the qualitative data collection methods adequate to address the research question? 1·3: Are the findings adequately derived from the data? 1·4: Is the interpretation of results sufficiently substantiated by data? 1·5: Is there coherence between qualitative data sources, collection, analysis and interpretation? 3·1. Are the participants representative of the target population? 3·2. Are measurements appropriate regarding both the outcome and intervention (or exposure)? 3·3. Are there complete outcome data? 3·4. Are the confounders accounted for in the design and analysis? 3·5. During the study period, is the intervention administered (or exposure occurred) as intended? 4·1. Is the sampling strategy relevant to address the research question? 4·2. Is the sample representative of the target population? 4·3. Are the measurements appropriate? 4·4. Is the risk of nonresponse bias low? 4·5. Is the statistical analysis appropriate to answer the research question? 5·1. Is there an adequate rationale for using a mixed methods design to address the research question? 5·2. Are the different components of the study effectively integrated to answer the research question? 5·3. Are the outputs of the integration of qualitative and quantitative components adequately interpreted? 5·4. Are divergences and inconsistencies between quantitative and qualitative results adequately addressed? 5·5. Do the different components of the study adhere to the quality criteria of each tradition of the methods involved?

**References**

?

80. Adhikary PK, Srivastava A, Kumar S, et al. Exploring the efficacy of large language models in summarizing mental health counseling sessions: benchmark study. JMIR Ment Health. Jul 23, 2024;11:e57306. [doi: 10.2196/57306] [Medline: 39042893]

31. Danner M, Hadzic B, Gerhardt S, et al. Advancing mental health diagnostics: GPT-based method for depression detection. Presented at: 2023 62nd Annual Conference of the Society of Instrument and Control Engineers (SICE); Sep 6-9, 2023; Tsu, Japan. [doi: 10.23919/SICE59929.2023.10354236]

**?**

65. Elyoseph Z, Levkovich I. Comparing the perspectives of generative AI, mental health experts, and the general public on schizophrenia recovery: case vignette study. JMIR Ment Health. 2024;11:e53043-e53043. [doi: 10.2196/53043]

98. Elyoseph Z, Levkovich I, Shinan-Altman S. Assessing prognosis in depression: comparing perspectives of AI models, mental health professionals and the general public. Fam Med Com Health. Jan 2024;12(Suppl 1):e002583. [doi: 10. 1136/fmch-2023-002583]

59. D’Souza RF, Amanullah S, Mathew M, Surapaneni KM. Appraising the performance of ChatGPT in psychiatry using 100 clinical case vignettes. Asian J Psychiatr. Nov 2023;89:103770. [doi: 10.1016/j.ajp.2023.103770] [Medline: 37812998]

82. Donnelly HK, Brown GK, Green KL, et al. Exploring the potential of large language models for automated safety plan scoring in outpatient mental health settings (preprint). JMIR Ment Health. Preprint posted online on Sep 3, 2025. [doi: 10.2196/preprints.79010] [Medline: 40196270]

47. Gargari OK, Fatehi F, Mohammadi I, Firouzabadi SR, Shafiee A, Habibi G. Diagnostic accuracy of large language models in psychiatry. Asian J Psychiatr. Oct 2024;100:104168. [doi: 10.1016/j.ajp.2024.104168]

?

86. He W, Zhang W, Jin Y, Zhou Q, Zhang H, Xia Q. Physician versus large language model chatbot responses to webbased questions from autistic patients in Chinese: cross-sectional comparative analysis. J Med Internet Res. Apr 30, 2024;26:e54706. [doi: 10.2196/54706] [Medline: 38687566]

?

75. Hodson N, Williamson S. Can large language models replace therapists? Evaluating performance at simple cognitive behavioral therapy tasks. JMIR AI. Jul 30, 2024;3:e52500. [doi: 10.2196/52500] [Medline: 39078696]

32. Hu C, Li W, Ruan M, et al. Exploiting ChatGPT for diagnosing autism-associated language disorders and identifying distinct features. Res Sq. May 21, 2024:rs.3.rs-4359726. [doi: 10.21203/rs.3.rs-4359726/v1] [Medline: 38826194]

44. Hur JK, Heffner J, Feng GW, Joormann J, Rutledge RB. Language sentiment predicts changes in depressive symptoms. Proc Natl Acad Sci U S A. Sep 24, 2024;121(39):e2321321121. [doi: 10.1073/pnas.2321321121] [Medline: 39284070]

76. Jain A, Sandhu R, Singh G, Rakhra M. The role of AI counselling in journaling for mental health improvement. Presented at: 2024 International Conference on Electrical Electronics and Computing Technologies (ICEECT); Aug 29-31, 2024:1-6; Greater Noida, India. [doi: 10.1109/ICEECT61758.2024.10739128]

?

?

?

?

51. Lauderdale SA, Schmitt R, Wuckovich B, Dalal N, Desai H, Tomlinson S. Effectiveness of generative AI-large language models’ recognition of veteran suicide risk: a comparison with human mental health providers using a risk stratification model. Front Psychiatry. 2025;16:1544951. [doi: 10.3389/fpsyt.2025.1544951] [Medline: 40248601]

36. Lee JJ, Han J, Woo CW. Interpretable depression assessment using a large language model. PLOS Digit Health. Feb 2026;5(2):e0001205. [doi: 10.1371/journal.pdig.0001205] [Medline: 41662257]

54. Levkovich I, Rabin E, Brann M, Elyoseph Z. Large language models outperform general practitioners in identifying complex cases of childhood anxiety. Digit Health. 2024;10:20552076241294182. [doi: 10.1177/20552076241294182] [Medline: 39687523]

?

?

64. Liu C, Wang WYC, Khan G. Understanding medical information and emotional support needs in mental health questions with large language models. Ind Manage Data Syst. Jun 22, 2026;126(7):2205-2230. [doi: 10.1108/IMDS-05-2025-0609]

33. Lorenzoni G, Velmovitsky PE, Alencar P, Cowan D. GPT-4 on clinic depression assessment: an LLM-based pilot study. Presented at: 2024 IEEE International Conference on Big Data (BigData); Dec 15-18, 2024:5043-5049; Washington, DC, USA. [doi: 10.1109/BigData62323.2024.10825184]

43. Marengo D, Longobardi C. Detecting suicidal ideation in adolescence using self-reported emotional and behavioral patterns: comparing machine learning and large language model predictions. Assessment. Dec 31, 2025:10731911251406405. [doi: 10.1177/10731911251406405] [Medline: 41472619]

84. Matsumura M, Nishida K, Toyoda K, et al. Quantifying improvement of psychotic symptoms in clozapine-treated schizophrenia: clinical note analysis with large language models. Sci Rep. Feb 13, 2026;16(1):8835. [doi: 10.1038/ s41598-026-39676-0] [Medline: 41680410]

69. McBain RK, Cantor JH, Zhang LA, et al. Evaluation of alignment between large language models and expert clinicians in suicide risk assessment. Psychiatr Serv. Nov 1, 2025;76(11):944-950. [doi: 10.1176/appi.ps.20250086] [Medline: 41174947]

91. McBain RK, Cantor JH, Zhang LA, et al. Competency of large language models in evaluating appropriate responses to suicidal ideation: comparative study. J Med Internet Res. Mar 5, 2025;27:e67891. [doi: 10.2196/67891] [Medline: 40053817]

39. McCoy TH, Perlis RH. Applying large language models to stratify suicide risk using narrative clinical notes. J Mood Anxiety Disord. Jun 2025;10:100109. [doi: 10.1016/j.xjmad.2025.100109] [Medline: 40657592]

40. McCoy TH, Perlis RH. Reasoning language models for more transparent prediction of suicide risk. BMJ Ment Health. May 11, 2025;28(1):e301654. [doi: 10.1136/bmjment-2025-301654] [Medline: 40350181]

?

?

?

?

67. Perlis RH, Goldberg JF, Ostacher MJ, Schneck CD. Clinical decision support for bipolar depression using large language models. Neuropsychopharmacology. Aug 2024;49(9):1412-1416. [doi: 10.1038/s41386-024-01841-2] [Medline: 38480911]

48. Sarma KV, Hanss KE, M Halls AJ, Becker DF, Glowinski AL, Krystal A. Simulated reasoning and self-verification in generalist large language models for psychiatric diagnostic performance: cross-sectional study. medRxiv. Preprint posted online on Sep 9, 2025. [doi: 10.1101/2025.09.05.25335196] [Medline: 40963745]

?

89. Senturk E, Koparal B. ChatGPT-4o vs psychiatrists in responding to common antidepressant concerns. Am J Health Promot. Jan 2026;40(1):10-17. [doi: 10.1177/08901171251348208] [Medline: 40440446]

79. Shen H, Li Z, Yang M, et al. Are large language models possible to conduct cognitive behavioral therapy? Presented at: 2024 IEEE International Conference on Bioinformatics and Biomedicine (BIBM); Dec 3-6, 2024:3695-3700; Lisbon, Portugal. [doi: 10.1109/BIBM62325.2024.10821773]

68. Silva R, Gomes L. An adaptive language model-based intelligent medication assistant for the decision support of antidepressant prescriptions. Comput Biol Med. May 2025;190:110065. [doi: 10.1016/j.compbiomed.2025.110065] [Medline: 40147190]

37. Shin D, Kim H, Lee S, Cho Y, Jung W. Using large language models to detect depression from user-generated diary text data as a novel approach in digital mental health screening: instrument validation study. J Med Internet Res. Sep 18, 2024;26:e54617. [doi: 10.2196/54617] [Medline: 39292502]

42. Sun M, Yu J, Long Z, et al. Large language models for psychiatric diagnosis based on multicenter real-world clinical records: comparative study. JMIR Med Inf. Jan 13, 2026;14:e77699. [doi: 10.2196/77699] [Medline: 41408781]

35. Teferra BG, Perivolaris A, Hsiang WN, et al. Leveraging large language models for automated depression screening. PLOS Digit Health. Jul 2025;4(7):e0000943. [doi: 10.1371/journal.pdig.0000943] [Medline: 40720397]

71. Thotapalli S, Yilanli M, McKay I, et al. Potential of ChatGPT in youth mental health emergency triage: comparative analysis with clinicians. PCN Rep. Sep 2025;4(3):e70159. [doi: 10.1002/pcn5.70159] [Medline: 40673126]

88. Yilanli M, et al. 2024.

?

30. Bartal A, Jagodnik KM, Chan SJ, Dekel S. AI and narrative embeddings detect PTSD following childbirth via birth stories. Sci Rep. Apr 11, 2024;14(1):8336. [doi: 10.1038/s41598-024-54242-2] [Medline: 38605073]

38. Bhagat N, Mackey O, Wilcox A. Large language models for efficient medical information extraction. AMIA Jt Summits Transl Sci Proc. 2024;2024:509-514. [Medline: 38827084]

49. Heston TF. Safety of large language models in addressing depression. Cureus. 2023;15(12):e50729. [doi: 10.7759/ cureus.50729]

?

50. Levkovich I, Elyoseph Z. Suicide risk assessments through the eyes of ChatGPT-3.5 versus ChatGPT-4: vignette study. JMIR Ment Health. Sep 20, 2023;10:e51232. [doi: 10.2196/51232] [Medline: 37728984]

53. Levkovich I, Elyoseph Z. Identifying depression and its determinants upon initiating treatment: ChatGPT versus primary care physicians. Fam Med Community Health. Sep 2023;11(4):e002391. [doi: 10.1136/fmch-2023-002391] [Medline: 37844967]

56. Levkovich I. Harnessing large language models for identification and treatment of obsessive-compulsive disorder. Comput Hum Behav: Artif Hum. Dec 2025;6:100212. [doi: 10.1016/j.chbah.2025.100212]

72. Acevedo S, Aneja E, Opler DJ, Valera P, Jarmon E. Evaluating the efficacy of ChatGPT-3.5 versus human-delivered text-based cognitive-behavioral therapy: a comparative pilot Study. Am J Psychother. Mar 1, 2026;79(1):4-11. [doi: 10.1176/appi.psychotherapy.20240070] [Medline: 40820507]

80. Adhikary PK, Srivastava A, Kumar S, et al. Exploring the efficacy of large language models in summarizing mental health counseling sessions: benchmark study. JMIR Ment Health. Jul 23, 2024;11:e57306. [doi: 10.2196/57306] [Medline: 39042893]

74. Haber Y, Levi-Belz Y, Elbak YS, Elyoseph Z, Levkovich I. Validating GenAI feedback in suicide prevention training: a mixed-methods study of QPR skill assessment. Front Med (Lausanne). 2025;12:1709743. [doi: 10.3389/fmed.2025. 1709743] [Medline: 41625774]

60. Jin KW, Rostam-Abadi Y, Chaudhary P, et al. Evaluating diagnostic accuracy and clinical reasoning of multiple large language models in psychiatry. medRxiv. Preprint posted online on Feb 11, 2026. [doi: 10.64898/2026.02.03.26345402] [Medline: 41728283]

58. Levkovich I, Haber Y, Levi-Belz Y, Elyoseph Z. A step toward the future? evaluating GenAI QPR simulation training for mental health gatekeepers. Front Med (Lausanne). 2025;12:1599900. [doi: 10.3389/fmed.2025.1599900] [Medline: 40568211]

87. Li Y, Wang G, Huang Z, et al. Development and preliminary evaluation of a virtual standardized patient system for psychiatric interview training. BMC Psychiatry. 2026;26(1):264. [doi: 10.1186/s12888-026-07896-3]

92. Scholich T, Barr M, Wiltsey Stirman S, Raj S. A comparison of responses from human therapists and large language model-based chatbots to assess therapeutic communication: mixed methods study. JMIR Ment Health. May 21, 2025;12:e69709. [doi: 10.2196/69709] [Medline: 40397927]

70. Tan KS, Cervin M, Leman P, Nielsen K, Kumar PV, Medvedev O. AI meets psychology: an exploratory study of large language models’ competence in psychotherapy contexts. J Psychol AI. Dec 31, 2025;1(1):2545258. [doi: 10.1080/29974100.2025.2545258]

52. Zeng Q, Li X, Wang S, Liu K. Adversarial evaluation algorithm for detecting extreme behaviors of llms in psychological counseling scenarios. Presented at: 2025 2nd International Conference on Algorithms, Software Engineering and Network Security (ASENS); Mar 21-23, 2025:412-415; Guangzhou, China. [doi: 10.1109/ ASENS64990.2025.11011189]

17. Zhang D, et al. Memory in large language models: mechanisms, evaluation and evolution. arXiv. Preprint posted online on 2025.
