# Supplementary material for: Generative Large Language Models in Mental Health Care Settings: Systematic Review and Meta-Analysis
Source: JMIR AI. 2026 Aug 31;5:e87730. doi: 10.2196/87730 (PMC13528826; doi:10.2196/87730)
Supplement: Supplementary file 2 [file ai-v5-e87730-s002.docx]

| **First Author, Year, Aim** | **Design, Setting, Sample Characteristics, Mental Health Condition** | **Intervention Details** | **LLM Application and Prompting Details** | **Comparator & Outcome Measures** | **Key Findings** |
| --- | --- | --- | --- | --- | --- |
| ***Screening and Diagnosis*** | | | | | |
| Bartal et al., 2024. [30] Aim: Evaluate ChatGPT for screening childbirth-related PTSD from narratives. | Retrospective: real-world online survey setting; no physical clinical setting; postpartum women (n=1295), self-reported childbirth narratives; childbirth-related PTSD | Written childbirth narratives from postpartum women were entered into the system. The model was asked to classify whether each narrative suggested childbirth-related PTSD. Different prompting setups were used for the same binary task, and each narrative was handled as a separate text case. | models: gpt-3.5-turbo-16k; text-embedding-ada-002 for embeddings; prompting info: zero-shot and few-shot prompting with explicit examples; evaluation date: data collected in 2016-2017 and 2020 | PCL-5 labels as the reference standard, with comparison against domain-specific pretrained language models and ChatGPT prompting variants; outcomes: F1 score, sensitivity, specificity, and AUC | For childbirth-related PTSD screening, ChatGPT performed poorly because sensitivity was low despite very high specificity. Zero-shot prompting achieved F1 0.33, sensitivity 0.20, and specificity 0.99, while few-shot prompting improved only slightly to F1 0.38, sensitivity 0.24, and specificity 0.96. These results suggest ChatGPT alone was not sufficient for clinical screening. |
| Bhagat et al., 2024 [38]; Identify depression from hospital clinical notes using an LLM | Retrospective; retrospective hospital EHR dataset, no live clinical deployment; mixed clinical patients, hospital clinical notes (n=100); Depression | Full clinical notes were provided to the LLM as input. The model was asked to determine whether specific conditions were present or absent in each note. Each record was processed independently using the same structured extraction task. The intervention involved condition identification from free-text clinical documentation. | models: GPT-3.5-Turbo prompting info: Structured extraction prompt for binary classification evaluation date: Data from 2022; evaluation date not explicitly reported | Manual human annotation by two reviewers with adjudication and physician validation served as the reference standard; sensitivity, specificity, and F1-score | The model identified depression with very high performance and fully consistent outputs across repeated runs. For depression, sensitivity was 1.00, specificity was 0.95, and F1-score was 0.89. Performance was also strong for other conditions including heavy smoking and cancer, but weaker for family history of heart disease. Sample size was 100 clinical notes. |
| D'Souza et al., 2023 [59]; Evaluate ChatGPT for psychiatric case-based clinical decision support | Vignette; simulated, no real clinical setting; patients with psychiatric disorders in textbook-derived clinical case vignettes (n=100); Psychiatric disorders | One hundred psychiatric case vignettes were entered into the LLM. The model was asked to complete clinical tasks including diagnosis, differential diagnosis, management, counselling, and clinical reasoning for each case. One initial response was recorded for each vignette without regeneration. Each vignette was treated as an independent simulated patient scenario. | models: ChatGPT-3.5 prompting info: Direct case vignette input without a structured prompt evaluation date: Study conducted in 2023 | Psychiatry textbook answers served as the reference standard; expert 0-10 ratings converted to grades A-D, including domain-specific performance such as diagnosis and management | ChatGPT-3.5 produced broadly acceptable responses across 100 psychiatric case vignettes. Overall, 61% of responses were graded A, 31% B, and 8% C, with no D grades. Performance was strongest for management tasks (43 A, 22 B, 6 C; n=71) and diagnosis tasks (30 A, 14 B, 4 C; n=48). Sample size was 100 vignettes. |
| Danner et al., 2023 [31]; Evaluate LLMs for transcript-based depression detection | Retrospective; retrospective dataset with simulated interviews, no live clinical setting; adults with and without depression, using clinical interview transcripts and simulated interviews from DAIC-WOZ; Depression | Transcribed clinical interviews were used as input data. The models were asked to classify whether each interview indicated depression. The same classification task was also applied to a simulated interview dataset. The intervention involved text-based depression detection from interview transcripts. | models: GPT-3.5; GPT-4 prompting info: Not clearly specified, likely zero-shot or few-shot classification evaluation date: Not explicitly reported | PHQ-8 depression labels served as the reference standard, with comparison across BERT, GPT-3.5, and GPT-4; precision, recall, and F1 score | GPT-3.5 showed the best LLM performance for depression detection on the DAIC-WOZ dataset, with precision 0.78, recall 0.79, and F1 0.78. GPT-4 performed worse on the same dataset (precision 0.70, recall 0.60, F1 0.61), while the best overall performance came from enhanced BERT trained on DAIC-WOZ plus Extended-DAIC (precision 0.83, recall 0.82, F1 0.82). Performance dropped notably on the simulated dataset. Sample sizes were 186 for DAIC-WOZ and 86 for E-DAIC. |
| Gargari et al., 2024 [47]; Evaluate LLM diagnostic accuracy on DSM-5 psychiatric vignettes | Vignette; simulated, no real clinical setting; patients with psychiatric disorders in DSM-5 clinical case vignettes (n=20); Psychiatric disorders | Clinical case vignettes based on DSM criteria were provided to the models. The models were asked to give the most likely diagnosis together with their reasoning. Some runs also supplied retrieved DSM text alongside the vignette. The intervention involved diagnosis generation from standardized psychiatric cases. | models: GPT-3.5 Turbo; GPT-4; Aya-101; Nemotron-3-8B prompting info: Zero-shot diagnostic prompting with optional DSM-5 retrieval augmentation evaluation date: 2024 | DSM-5 case diagnoses served as the reference standard; number of correct diagnoses, proportions of partially correct and incorrect responses, and reasoning quality categories | GPT-3.5 Turbo and GPT-4 showed the best diagnostic accuracy, each correctly identifying 13 of 20 cases (65%), while GPT-3.5 CA achieved 60%, Nemotron 35%, and Aya 30%. GPT-4 had one incorrect diagnosis, whereas GPT-3.5 had none. Performance was weaker for temporally nuanced disorders such as cyclothymic disorder, disruptive mood dysregulation disorder, peripartum depression, and acute stress disorder. |
| Heinz et al., 2023 [46]; Evaluate GPT-3 diagnostic accuracy across psychiatric vignettes | Vignette; simulated, no real clinical setting; patients with psychiatric disorders in DSM-5-based simulated vignettes (n=59, expanded outputs n=1710); Psychiatric disorders | Researchers created structured psychiatric vignettes and systematically varied demographic features within them. The model was given small sets of example cases and then asked to diagnose unlabeled cases. Each vignette was processed as a separate diagnostic task. The intervention examined diagnosis generation across standardized cases with demographic variation. | models: GPT-3 (175B parameters) prompting info: Few-shot prompting with two labeled and four unlabeled examples; temperature 0.5-0.55 and max tokens 300 evaluation date: Not explicitly reported (submitted June 2022; accepted March 2023) | DSM-5-based correct diagnoses for each vignette served as the reference standard; balanced accuracy, sensitivity, specificity, PPV, NPV with 95% CI, and mixed-effects models assessing demographic bias | Diagnostic performance varied by condition, with balanced accuracy ranging from 0.50 to 0.82 across 1,710 model responses. Highest performance was seen for ADHD (0.82) and PTSD (0.81), while bipolar disorder (0.58), somatic symptom disorder (0.54), and functional neurological disorder (0.50) performed worst. The model generally showed higher specificity and negative predictive value than sensitivity and positive predictive value, suggesting stronger rule-out than detection performance; bias analyses also found significant ethnicity- and sex-based differences for selected diagnoses. |
| Heston, 2023 [49]; Evaluate LLM responses to escalating depression and suicidality prompts | Vignette; simulated, no real clinical setting; LLM mental health interactions (n=25 agents) in simulated conversations; Depression and suicidality | Simulated patient scenarios involving escalating suicidality or depressive symptoms were entered into a set of LLM agents. Each agent received the prompts in a fixed sequence without deviation. Researchers tracked how conversations progressed as risk level increased. The intervention tested LLM behavior during escalating mental health crisis prompts. | models: ChatGPT-3.5 prompting info: Pre-structured conversational agent prompts from the FlowGPT repository evaluation date: September 2023 | No direct human comparator; reference standard was expected safe clinical behavior for escalation decisions; prompt number at initial referral to human, prompt number at conversational shutdown, PHQ-9-derived severity score, crisis resource referral, and restart behavior after shutdown | Most LLM agents referred users onward only after symptoms had escalated to moderate depression severity and typically shut down only near severe crisis levels. Initial referral occurred at mean prompt 3.92 (approximate PHQ-9 score 12), while shutdown occurred at mean prompt 8.32 (PHQ-9 about 25-27). Only 8% provided suicide hotline resources, and 88% resumed conversation after shutdown, indicating important safety limitations. |
| Hu et al., 2024 [32]; Evaluate LLM-based ASD language assessment from transcripts | Retrospective; retrospective recorded interview dataset, no live clinical setting; adults with ASD (n=44) using structured diagnostic interaction transcripts from the ADOS dataset; Autism spectrum disorder | Audio recordings from structured autism assessments were transcribed into examiner-patient dialogue. The LLM received these transcripts and was asked to judge the presence of social language disorder and identify specific language features. Each scenario contributed transcript-based evidence for the final output. The intervention involved transcript-based language-feature assessment in ASD. | models: ChatGPT (exact version not reported; likely GPT-3.5 or GPT-4 API) prompting info: Structured prompts combining dialogue, question text, and domain knowledge in a zero-shot classification framework evaluation date: Study posted May 2024 | ADOS-2 A4 scoring labels served as the reference standard, with comparison against BERT, RoBERTa, XLNet, ALBERT, and DistilBERT; accuracy, precision, recall, and F1 score for binary classification of ASD-related language disorder | ChatGPT substantially outperformed the best BERT baseline for ASD language assessment from transcript data. Accuracy was 81.82%, precision 82.45%, recall 81.82%, and F1-score 79.89%, compared with 63.92% accuracy and 61.87% F1 for BERT. This reflected clear improvement over conventional transformer baselines in this small ASD transcript dataset. |
| Hur et al., 2024 [44]; Evaluate LLM-derived sentiment for predicting depression severity | Prospective; real-world online study setting, no physical clinical setting; general population adults (n=467) providing survey responses and sentiment ratings; Depression | Participants completed brief open-ended writing tasks about mood-related experiences. The text responses were then entered into the LLM for sentiment scoring. The resulting sentiment values were used as a language-based representation of mood. The intervention involved LLM analysis of short written responses rather than direct therapeutic interaction. | models: ChatGPT (GPT-3.5 and GPT-4) prompting info: Structured prompts requesting numerical sentiment ratings from 0 to 10 evaluation date: Not explicitly stated | Human raters served as the main reference, with LIWC and baseline PHQ-9 scores used as additional comparators; prediction of 3-week change in PHQ-9 using robust linear regression, Spearman correlation, cross-validation, RMSE, MAE, and Wald test model comparisons | More negative language was associated with greater current and future depression across both studies, and ChatGPT-derived sentiment closely tracked human ratings. Correlations with current depression were ρ=-0.66 and -0.58, and with future depression were ρ=-0.61 and -0.60. ChatGPT sentiment significantly predicted increased depression at 3 weeks, whereas LIWC did not, suggesting added predictive value for short-term depression risk. |
| Jin et al., 2026 [60]; Evaluate frontier LLMs on different psychiatric diagnosis reasoning | Vignette; simulated, no real clinical setting; patients with psychiatric disorders in clinical case vignettes (n=196); Psychiatric disorders | Psychiatric vignette text was entered into the models in a zero-shot format. The models were asked to generate and rank the top differential DSM diagnoses and explain their reasoning. Each vignette was processed as a standalone diagnostic case. The intervention involved multi-diagnosis generation from standardized psychiatric cases. | models: Claude Opus 4.5; Gemini 3 Pro; GPT-5.2; DeepSeek-V3.2 prompting info: Zero-shot, role-based prompting with structured instructions and explicit reasoning-trace requests evaluation date: December 2025 to January 2026 | DSM-5-TR vignette diagnoses served as the reference standard, with psychiatry residents included as an additional comparator on a subset; top-1 accuracy, top-5 accuracy, Recall@5, MRR, clinician-rated reasoning scores, and mixed-effects logistic regression linking reasoning quality to correctness | Across 196 psychiatric vignettes, top-1 accuracy ranged from 0.566 to 0.638 and top-5 accuracy from 0.730 to 0.801, with Claude Opus 4.5 performing best. Better reasoning quality was strongly associated with correct diagnosis, corresponding to about a sixfold increase in odds of correctness per one-point increase in reasoning score. Model performance appeared comparable to psychiatry residents on illustrative comparisons. |
| Kaliosis et al., 2026; Estimate PTSD severity from self-recorded clinical interviews | Retrospective; real-world clinical program setting at the Stony Brook World Trade Center Health and Wellness Program; adults completing self-recorded interviews and PTSD assessments (n=1,437); PTSD | LLMs were applied to transcribed clinical interview narratives to estimate PTSD severity scores. Prompts varied across zero-shot, few-shot, contextual, and reasoning-effort strategies, with post-hoc redistribution and ensemble approaches also tested. | models: LLaMA-3.1 (8B, 70B, 405B); DeepSeek-R1; GPT-5; o3-mini; 4o-mini; gpt-oss prompting info: Zero-shot, few-shot, chain-of-thought/TSBS, and varying reasoning effort evaluation date: Not reported | Human-rated subset and supervised models served as comparators; Pearson correlation, MAE, and bootstrap significance testing | GPT-5 was the best individual model, with correlation 0.475 and MAE about 7.95, while LLaMA-3.1-70B also performed strongly. Contextual prompting improved performance, higher reasoning effort helped some models, and GPT-5 outperformed human raters in the human-rated subset. The best overall result came from an ensemble with a supervised model. |
| Levkovich & Elyoseph, 2023a [50]; Compare LLM suicide-risk judgments with professionals | Vignette; simulated, no real clinical setting; patients with suicidality in simulated vignette scenarios; Suicide risk | Suicide-related vignettes were presented to the models. The models rated psychache, suicidal ideation, suicide attempt likelihood, and resilience using rating scales. The intervention examined suicide-risk-related judgment from standardized cases. | models: ChatGPT-4; ChatGPT-3.5 prompting info: Standardized vignette with structured Likert-scale questions evaluation date: June to July 2023 | Mental health professionals served as the comparator; Z-score deviation from professional norms, mean Likert scores, and comparisons across vignette conditions | ChatGPT-4 was closest overall to professional judgment but tended to overestimate some risks, while ChatGPT-3.5 more consistently underestimated risk. Both models underestimated resilience. ChatGPT-4 aligned most closely with professionals for suicide attempt risk. |
| Levkovich et al., 2024 [54]; Compare LLM recognition of childhood anxiety disorders | Vignette; simulated, no real clinical setting; children with anxiety disorders in simulated pediatric vignettes (n=5); Childhood anxiety disorders | Pediatric anxiety vignettes were entered into the models in separate sessions. The models identified the main complaint, a secondary complaint, and referral destination. The intervention focused on symptom recognition and referral planning. | models: ChatGPT-3.5; ChatGPT-4; Claude; Gemini prompting info: Standardized vignette with a structured questionnaire evaluation date: February 2024 | General practitioners and mental health professionals served as comparators; first and second identification, total recognition, and referral appropriateness | Claude and Gemini showed the highest recognition rates at 78%, outperforming both human groups and ChatGPT models. Referral recommendations differed substantially, with LLMs favoring mental health referral more often than GPs. Group differences were significant across recognition outcomes. |
| Levkovich, 2025 [56]; Compare LLM responses across multiple psychiatric vignettes | Vignette; simulated, no real clinical setting; patients with psychiatric disorders in DSM-5/ICD-11 vignette set (n=6); Depression, suicidality, schizophrenia, social phobia, PTSD | Six psychiatric vignettes were entered into the models. The models identified diagnosis, recommended treatment, and predicted outcomes with and without treatment. Each vignette was repeated multiple times for each model. | models: Gemini 2.0 Flash Experimental; Claude 3.5 Sonnet; ChatGPT-3.5; ChatGPT-4 prompting info: Standard vignette input without additional prompt engineering evaluation date: May 2024 | Mental health professionals from prior benchmark studies served as comparators; diagnostic accuracy, treatment recommendation frequency, and outcome prediction distributions | Diagnostic performance was high for common conditions such as depression, social phobia, and PTSD, often reaching 100% accuracy. Performance was weaker and more variable for schizophrenia. LLMs generally offered broader treatment recommendations but were more pessimistic than professionals about prognosis. |
| Shin et al., 2024 [37]; Evaluate diary-based depression detection using LLMs | Retrospective; real-world digital app setting on a community diary platform; general population adults (n=91) with diary entries (n=428); Depression | Users wrote daily emotional diary entries in a mobile app. The text was analyzed by LLMs to classify entries as depressed or non-depressed. Several prompting and fine-tuning approaches were applied to the same task. | models: GPT-3.5; GPT-4 prompting info: Zero-shot and chain-of-thought prompting evaluation date: Data collected October 2022 to April 2023 | PHQ-9 and BSS cutoffs plus psychiatrist adjudication served as the reference standard; cross-validated classification performance | GPT-3.5 had the best balanced accuracy at 0.844 and recall 0.929, while GPT-4 achieved the highest recall but lower overall accuracy and F1. Findings suggested a trade-off between balanced performance and maximal sensitivity. |
| Jin et al., 2024 [45]; Evaluate a GPT-based conversational depression screening tool | Prospective; simulated screening assessment, no real clinical setting; adults with depression (n=20) undergoing interview and diagnostic assessment; Depression | A custom GPT system delivered an open-ended BDI-Fast Screen, asking structured depression-screening questions one by one and scoring free-text answers semantically. The intervention was an interactive GPT-based depression assessment. | models: Custom ChatGPT (GPTs) prompting info: Structured multi-step prompts replicating the BDI-FS conversationally evaluation date: Created June 7, 2024 | Psychiatrist clinical diagnosis using CIS-R and ICD-10 served as the reference standard, with PHQ-9 as additional comparator; correlation, agreement, AUC, sensitivity, and false-positive rate | The GPT-based BDI-Fast Screen showed excellent agreement with diagnosis and outperformed PHQ-9 in this small sample. Correlation with PHQ-9 was very high, agreement with diagnosis was stronger than PHQ-9, and classification AUC reached 1.00. Authors noted likely overestimation due to small sample size. |
| Lauderdale et al., 2025 [51]; Compare LLM and provider suicide-risk ratings in veteran vignettes | Vignette; simulated, no real clinical setting; standardized veteran cases with repeated evaluations; Suicide risk | LLMs were given veteran vignettes plus the VHA suicide risk stratification table. They rated acute and chronic suicide risk and recommended treatment disposition. | models: ChatGPT-3.5; ChatGPT-4o; Google Gemini prompting info: Zero-shot structured prompting using the VHA table evaluation date: July 23-24, 2024 | Ratings from human mental health care providers served as comparators; t-tests, ANOVA, regression, effect sizes, means, and SDs | LLMs approximated human suicide-risk ratings but showed systematic biases and inter-model variability. They underrated the highest-risk vignette and overrated the lowest-risk vignette for acute risk. ChatGPT-4o recommended hospitalization for all cases, and chronic risk more strongly predicted disposition than acute risk. |
| Lee et al., 2026 [36]; Estimate depression severity from interview transcripts using LLM features | Retrospective; retrospective virtual interview transcript dataset, no live clinical setting; adults with and without depression using DAIC-WOZ/E-DAIC transcripts (n≈272 total); Depression | Semi-structured interview transcripts were used as input. An LLM assigned numeric ratings to depression-related symptom, language, and cognitive-distortion factors, which were then used to estimate severity. | models: Meta LLaMA 3.1-70B-Instruct prompting info: Zero-shot prompting asking one depression-related question at a time with 0-10 scoring evaluation date: Not explicitly reported | PHQ-8 depression severity scores served as the main reference standard, with prior text-based models and other feature sets as comparators; MAE, RMSE, R², and correlations | LLM-derived features predicted depression severity well, with MAE 2.86-2.91 and R² 0.63-0.66, outperforming prior text-based models. Clinical symptom features were most informative, especially depressed mood, negative sentiment, and sleep disturbance. |
| Lho et al., 2025 [41]; Predict depression and suicide risk from sentence completion narratives | Retrospective; real-world psychiatric clinical setting at Seoul Metropolitan Government-Seoul National University Boramae Medical Center; depression and suicide-risk cases from sentence completion narratives; Depression and suicide risk | Deidentified sentence completion test narratives across multiple domains were concatenated and entered into LLMs. Models classified each patient as high or low risk for depression and suicide using zero-shot and few-shot prompting. | models: GPT-4o; Gemini 1.0 Pro; GPT-3.5-turbo-16k prompting info: Zero-shot and few-shot prompting evaluation date: July 4 to September 30, 2024 | Ground-truth labels from BDI-II, Zung SDS, and BSS served as reference, with embedding-based ML models as comparators; AUROC, accuracy, macro F1, and statistical comparisons | LLM performance was moderate and improved with few-shot prompting, especially for GPT-4o and Gemini. Self-concept narratives gave the strongest predictive performance. Overall, embedding-based models outperformed the LLMs for both depression and suicide-risk prediction. |
| Liu et al., 2025 [64]; Extract medical and emotional needs from mental health questions | Retrospective; simulated questions from online consultation platforms (n=703 questions); General mental health | LLMs processed mental health questions using a structured joint prompt. They identified medical needs, extracted related clinical context, identified emotional needs, and extracted emotional causes, returning structured span annotations. | models: GPT-4o; DeepSeek-V3; DeepSeek-R1; Qwen2.5-Max; Qwen2.5-72B; Qwen3-235B prompting info: Joint prompt combining chain-of-thought and few-shot prompting evaluation date: Not reported | Human-annotated gold-standard MHQ-MedEmo dataset served as the reference standard; precision, recall, F1 by task, joint exact match, latency, and span matching | On the 141-query test set, DeepSeek-R1 had the best overall non-fine-tuned performance with F1 0.693, but much slower latency. Models performed better on structured medical tasks than subjective emotional tasks. Joint prompting improved both accuracy and efficiency compared with separate prompts. |
| Lorenzoni et al., 2024 [33]; Test prompt effects on transcript-based depression detection | Retrospective; retrospective clinical interview transcript dataset, no live clinical setting; adults with and without depression using DAIC-WOZ transcripts (n=100 sessions); Depression | Interview transcripts were entered for binary depression classification. Multiple prompting strategies were tested, including simple, few-shot, and clinically framed prompts, with different temperature settings. | models: GPT-4 via OpenAI API prompting info: Zero-shot, few-shot, and complex clinical prompting; temperature 0.0-0.5 evaluation date: Not explicitly reported | DAIC-WOZ PHQ-8 depression labels served as the reference standard; accuracy, precision, recall, F1, and confusion matrices | Prompting strategy strongly affected performance. Few-shot prompting markedly improved recall, while low-temperature complex prompting gave the best overall performance, with peak accuracy 73.37% and F1 63.70%. Higher temperatures reduced reliability. |
| Marengo & Longobardi, 2025 [43]; Estimate adolescent suicidal ideation from questionnaire data | Retrospective; real-world school-based screening setting; adolescents (n=1,197) with self-report questionnaire data; Adolescent suicidal ideation | Structured SDQ responses and demographic information were given to the models. The models estimated a continuous suicidal ideation score, with repeated querying and averaged outputs. | models: GPT-4o; Gemini 1.5 Pro prompting info: Structured clinical reasoning simulation with numeric score output only, repeated sampling at k=3, temperature 0.5 evaluation date: Not explicitly reported | SIQ-JR scores served as the reference standard, with ML models based on structured data and LLM outputs as comparators; correlations, ROC AUC, sensitivity, and specificity | LLM-derived scores correlated moderately with SIQ-JR and achieved AUCs around 0.83-0.85. Traditional SDQ-based machine-learning models slightly outperformed LLM-based models. Reliability was high, especially for Gemini and GPT-4o, but LLMs showed a conservative bias. |
| McCoy & Perlis, 2025 [39]; Predict suicide death risk from discharge summaries | Retrospective; real-world inpatient hospital setting; adults (n=11,954) in hospital discharge EHR dataset; Suicide risk and mortality | Narrative discharge summaries were entered into a secure GPT-based system. The model estimated a continuous probability of future suicide death hazard using structured risk and protective factor framing. | models: GPT-4-1106-preview via Azure OpenAI prompting info: Zero-shot prompting with structured clinical guidance and temperature 0 evaluation date: Not explicitly reported | A baseline model using only structured demographic and clinical variables served as comparator; hazard ratios, ROC AUC, calibration, and survival differences | LLM-derived discharge-summary risk scores were strongly associated with later suicide or accidental death. Discrimination was modest for the primary outcome but better for suicide-only prediction, and survival differed clearly across risk quartiles. Calibration improved substantially after recalibration. |
| McCoy & Perlis, 2025B [40]; Compare distilled and larger models for suicide-risk prediction | Retrospective; real-world inpatient hospital setting; adults in hospital discharge EHR dataset (n=11,954); Suicide risk | Raw hospital discharge summaries were entered directly into the model. The model estimated 12-month suicide-risk probability and returned structured outputs including risk and protective factors. | models: DeepSeek-R1-Distill-Llama-8B; Llama 3.1 8B; Llama 3.2 3B; GPT-4o prompting info: Zero-shot structured prompting with psychiatrist role and risk-factor framing evaluation date: Not explicitly stated | Prior GPT-4o results and other Llama models served as comparators; C-statistic, hazard ratios, Kaplan-Meier analysis, and calibration | In 11,954 discharges, GPT-4o outperformed the distilled Llama model for suicide-risk prediction, but the distilled model performed only slightly worse. Risk scores were significantly associated with outcomes, suggesting reasonable portability at lower computational cost. |
| Sarma et al., 2025A [48]; Test self-verification for DSM-5-TR vignette diagnosis | Vignette; simulated, no real clinical setting; patients with psychiatric disorders in DSM-5-TR clinical case vignettes (n=93 total, 55 test cases); Psychiatric disorders | Standardized DSM-5-TR case vignettes were entered into several models. Models either generated diagnoses directly or rechecked them through pairwise self-verification prompts. | models: gpt-4.1; o3-pro; Gemini 2.5 Flash; Gemini 2.5 Pro prompting info: Basic diagnosis prompting plus self-verification pairwise post-processing evaluation date: Not explicitly reported | Author-designated DSM-5-TR Clinical Cases diagnoses served as the reference standard; sensitivity, PPV, and mixed-effects models | Across 106 vignette assessments, o3-pro achieved the strongest overall performance. Self-verification significantly improved PPV, and large reasoning models performed better than standard LLMs. No fixed effects significantly improved sensitivity. |
| Sarma et al., 2025B; Compare direct and decision-tree prompting for psychiatric diagnosis | Vignette; simulated, no real clinical setting; patients with mental health conditions using LLM interaction logs (n=3 systems, 2 scenarios); Psychiatric disorders | Narrative psychiatric case vignettes were entered into GPT models using two methods. In the direct method, the model returned diagnoses straight from the vignette. In the decision-tree method, the model was guided through iterative yes/no diagnostic branches before narrowing the diagnosis. The intervention compared direct prompting with structured decision-tree questioning. | models: gpt-3.5-turbo-0125; gpt-4-0125-preview; gpt-4o-2024-05-13 prompting info: Base direct prompting versus expert-guided decision-tree iterative prompting with a pairwise comparison step evaluation date: August 2024 | There was no external comparator beyond comparison of base versus decision-tree prompting; PPV, sensitivity, and F1 at both diagnosis and diagnostic-category levels, with paired two-tailed Student’s t-tests comparing base versus decision-tree prompting | On 55 DSM-5-TR test vignettes with 77 author-designated diagnoses, decision-tree prompting improved PPV and usually improved F1, but at the cost of reduced sensitivity. The best base model was GPT-4o, and expert decision trees significantly increased PPV in all experiments and improved F1 in five of six. |
| Sun et al., 2026 [42]; Evaluate inpatient psychiatric diagnosis from admission narratives | Retrospective; retrospective multicenter inpatient psychiatric EHR dataset; psychiatric inpatients (n=9,923); Psychiatric disorders | Deidentified Chinese inpatient psychiatric narratives were entered into general-purpose LLMs. The models generated the most likely diagnosis from the admission narrative alone using the same prompt for each case. | models: GPT-4.0; GPT-3.5; GLM-4-Plus prompting info: Zero-shot inference with one standardized prompt evaluation date: January 2025 | Physician-confirmed discharge diagnoses served as the reference standard; exact-match accuracy, precision, recall, F1, and ordinal regression | GPT-4 achieved the highest diagnostic accuracy at 71.7% and the highest weighted F1, significantly outperforming GPT-3.5. Performance was higher in older adults, lower in adolescents, and stable across centers. |
| Teferra et al., 2025 [35]; Score PHQ-8 symptoms from depression interview transcripts | Retrospective; retrospective virtual interview dataset, no live clinical setting; psychiatric outpatients (n=1,160) with speech and EHR diagnostic data; Depression | Deidentified interview transcripts were linked to item-level PHQ-8 labels. Models identified evidence for each symptom and assigned Likert or binary symptom scores. | models: GPT-3.5; GPT-4o; GPT-4 Turbo; GPT-4o Mini; Cohere Command R+; Llama-3-8B-Instruct; Gemini-1.5-flash prompting info: Zero-shot prompting using the RISEN framework evaluation date: Not explicitly reported | DAIC-WOZ PHQ-8 item labels served as the reference standard, with models compared against each other; accuracy, precision, recall, F1, and MCC | GPT-4o showed the best overall performance for binary symptom scoring, with accuracy 75.9% and F1 0.74. Likert scoring was notably lower. GPT models performed best on emotional and cognitive symptoms, though some non-GPT models performed best for selected items. |
| Zeng et al., 2025 [52]; Test LLM stability in recognizing high-risk behaviours | Vignette; simulated, no real clinical setting; 1,812 text cases built from EmoLLM and Weibo posts; High-risk behaviours | Counseling-style text cases were entered into the models, then adversarially modified while preserving risky behavior. The intervention examined whether recognition and intervention responses remained stable across contextual changes. | models: DeepSeek; GPT-4; Claude; Wenxin Yiyan prompting info: Not explicitly reported evaluation date: Not explicitly reported | Models were compared against each other without a human gold standard; intervention rate, Behavior Sensitivity Index, Consistency Rate, and chi-square tests | Across 1,812 cases, Claude had the highest behavior sensitivity, while DeepSeek had the highest consistency. GPT-4 intervened least often and showed the greatest inconsistency, indicating variability in both willingness and stability of intervention. |
| ***Clinical Decision Support*** | | | | | |
| Dergaa et al., 2024; Evaluate an LLM-enabled virtual psychiatric patient for trainee interview training | Prospective; simulated, no real clinical setting; psychiatric patients in simulated cases with trainee-led clinical interactions (n=20 participants); Psychiatric disorders | Trainees interacted with a virtual standardized patient system in spoken psychiatric scenarios. The system simulated history taking, mental state examination, and discussion of diagnosis and treatment using predefined patient personas. Learners practiced with the virtual patients as part of a structured teaching block. The intervention was LLM-enabled psychiatric interview training. | models: ERNIE 3.5-8K (Baidu) prompting info: Structured persona-based prompting with demographics, symptoms, and behavior rules, followed by iterative refinement evaluation date: Development and Delphi process from January to April 2024; trial timing not explicitly reported | Traditional instructor-led teaching with discussion served as the comparator; OSCE clinical interview skills score, user experience (UEQ), presence (IPQ), repeated-measures ANOVA, and mean differences | The system showed broadly strong performance overall, with 61% of responses graded A, 31% graded B, and 8% graded C; no responses were graded D. Performance was strongest in management (43 A, 22 B, 6 C; n=71) and diagnosis tasks (30 A, 14 B, 4 C; n=48), indicating good capability in core psychiatric training tasks without any completely incorrect responses. |
| Elyoseph & Levkovich, 2024 [65]; Compare LLM responses to schizophrenia case vignettes | Vignette; simulated, no real clinical setting; patients with schizophrenia in simulated case vignettes (n=2, repeated evaluations); Schizophrenia | Standardized schizophrenia vignettes were entered into the models. The models were asked to judge diagnosis, treatment, prognosis, long-term outcomes, and discrimination risk. Each vignette was run repeatedly in separate sessions. The intervention involved repeated response generation for simulated schizophrenia cases. | models: ChatGPT-3.5; ChatGPT-4; Bard; Claude prompting info: Standardized vignette with a fixed six-question questionnaire evaluation date: August 2023 | Responses were compared with existing datasets from mental health professionals and the general public; distribution of categorical prognosis outcomes, long-term outcome scores on a −5 to +5 scale, and statistical differences between LLMs and humans | All four LLMs correctly identified schizophrenia and recommended antipsychotic medication plus psychotherapy. ChatGPT-4, Bard, and Claude showed the closest agreement with mental health professionals, whereas ChatGPT-3.5 was consistently more pessimistic about prognosis and long-term outcomes. All models predicted worsening without treatment and anticipated discrimination, but ChatGPT-3.5 was notably more negative. |
| Elyoseph et al., 2024 [98]; Compare LLM responses to depression case vignettes | Vignette; simulated, no real clinical setting; patients with depression in simulated case vignettes (n=2, repeated evaluations); Depression | Standardized depression vignettes were entered into the models. The models were asked to identify the diagnosis, recommend treatment, and predict outcomes with and without care. They also responded to questions about longer-term consequences and discrimination. The same vignettes were run multiple times in separate sessions. | models: ChatGPT-3.5; ChatGPT-4; Claude; Bard prompting info: Standardized vignette with a structured six-question questionnaire evaluation date: August 2023 | Responses were compared with prior validated survey responses from mental health professionals and the general public; distribution of prognosis categories, long-term outcome scores on a −5 to +5 scale, and group comparisons using ANOVA | All LLMs correctly identified depression and recommended psychotherapy plus antidepressants, but prognosis varied across models. ChatGPT-4 more often predicted full recovery with recurrence, Bard was most optimistic, Claude gave mixed forecasts, and ChatGPT-3.5 was most pessimistic, often predicting only partial recovery. Without treatment, all models predicted no improvement or worsening, with significant between-model differences both with and without treatment. |
| Kim and Wang, 2026; Translate ICD-11 mood-disorder criteria into executable logic | Vignette; simulated, no real clinical setting; simulated patient cases derived from ICD-11 CDDR criteria (n=30 cases) with structured symptom and clinical history inputs; Mood disorders | LLMs were used to translate ICD-11 diagnostic criteria from natural language into Datalog logic programs. These generated rules were then executed in a constraint logic programming engine to produce diagnoses from structured patient symptom and history inputs. The workflow included one-shot generation of candidate logic rules, optional expert review and correction, and automated diagnosis using the finalized program. | models: GPT-4o; Gemini-1.5-Flash; LLaMA-3.2 prompting info: One-shot in-context prompting for translation of diagnostic text into Datalog, with separate prompting for direct diagnosis evaluation date: October to November 2024 | Gold-standard diagnoses based on ICD-11 CDDR criteria, manually validated; additional comparison against direct LLM diagnosis and LLM-generated logic programs without expert correction; diagnostic accuracy against the known diagnosis, with qualitative error analysis | Across 30 simulated patient cases, direct LLM diagnosis was highest for GPT-4o with 22/30 correct. Uncorrected LLM-generated logic programs performed similarly for GPT-4o but substantially worse for Gemini and LLaMA-3.2. After expert review and correction of the generated logic rules, the final logic-program system achieved 30/30 correct diagnoses, though substantial human editing was required. |
| Kim et al., 2025; Evaluate LLM diagnostic accuracy for OCD case vignettes | Vignette; simulated, no real clinical setting; patients with OCD and other disorders in clinical case vignettes (n=26); Obsessive-compulsive disorder | Clinical vignettes describing OCD and non-OCD cases were entered into the models. The models were asked to provide their top diagnoses, rank them, and explain their reasoning. Each vignette was run in a fresh session. The intervention was diagnostic interpretation of OCD-related case vignettes. | models: ChatGPT-4, Gemini Pro, Llama 3 prompting info: Zero-shot prompting evaluation date: April 2023 to April 2024 model versions reported | Human performance from prior vignette-based studies served as the comparator; proportion with OCD ranked top, control accuracy, and Cohen’s kappa agreement | Overall diagnostic accuracy was 96.1%, with ChatGPT-4 achieving 100% accuracy on OCD vignettes. All LLMs correctly classified control vignettes and outperformed all human comparator groups, with perfect agreement overall. Some models refused sexual-content vignettes due to safety filters. |
| Levkovich, 2024; Evaluate LLM diagnosis and treatment recommendations for OCD | Vignette; simulated, no real clinical setting; patients with OCD in validated clinical vignettes (n=6, repeated evaluations); Obsessive-compulsive disorder | OCD and control vignettes were entered with standardized prompts. Models selected diagnoses, rated confidence, chose treatments, and rated treatment confidence, and were also asked about stigma-related harm and willingness to engage. | models: ChatGPT-3.5-turbo; GPT-4; Claude 3.5 Sonnet; Gemini 1.5 Pro prompting info: Standardized vignette with a fixed questionnaire evaluation date: March 2024 | Mental health professionals served as the comparator; recognition rate, treatment recommendation rate, confidence, stigma measures, and chi-square comparisons | LLMs achieved markedly higher diagnostic accuracy than human comparators, with recognition rates of 90-100% versus 47.3% for humans. They more often recommended evidence-based treatment, showed greater confidence, and showed no perceived-danger bias. Group differences were strongly significant. |
| McBain et al., 2025A [69]; Evaluate LLM scoring of suicide-intervention responses | Vignette; simulated, no real clinical setting; patients with suicidality in hypothetical response scenarios (SIRI-2, n=24 scenarios); Suicidality | The models were given the original SIRI-2 instructions together with vignette scenarios and paired clinician responses. For each response, the models were asked to score how appropriate it was on the SIRI-2 scale. The same standardized instructions were used across models. The intervention involved rubric-based rating of suicide-intervention responses. | models: ChatGPT-4o; Claude 3.5 Sonnet; Gemini 1.5 Pro prompting info: Standardized instructions only, without additional prompt engineering evaluation date: June to July 2024 | Expert suicidologists from the original SIRI-2 validation study served as the benchmark comparator; mean difference in appropriateness scores versus experts, correlation with expert ratings, z-score deviations, final SIRI-2 score, and percentage of statistically significant outliers | All three models showed upward bias when rating suicide-intervention responses. Claude 3.5 performed best overall, with the highest correlation with experts (r=0.96) and the lowest outlier rate (11%), while Gemini performed worst with the highest outlier rate (36%). ChatGPT-4o performed around master’s-level counselor performance, whereas Gemini more closely resembled untrained school staff. |
| Nyugen et al., 2025; Benchmark LLMs on mental health counseling exam questions | Vignette; simulated, no real clinical setting; multiple-choice counseling benchmark questions derived from NCMHCE mock exam case studies (n=1612 questions from 138 case studies), covering 51 mental health conditions; General mental health | LLMs were evaluated on case-based multiple-choice counseling questions derived from mock counseling exam scenarios. Each question included patient context such as demographics, symptoms, and clinical history, together with multiple answer options. Models were tested using zero-shot, few-shot, chain-of-thought, and self-consistency prompting. Outputs were compared with correct answers and expert rationales. | models: GPT-4o; Llama-2; Llama-3; Meditron; Med42; OpenBioLLM; ClinicalCamel; Asclepius; BioMedGPT; MentaLLaMA; and other benchmarked models prompting info: Zero-shot, few-shot, chain-of-thought, and self-consistency evaluation date: Not reported | Ground-truth answers from the NCMHCE-derived benchmark, with expert-generated rationales; overall and competency-level accuracy, passing threshold at 63% accuracy, reasoning quality metrics, and ROSCOE metrics | GPT-4o achieved the highest overall accuracy at 0.78, while the best open-source model reached 0.717; several frontier models exceeded the 63% passing threshold. Performance was strongest on Intake, Assessment and Diagnosis, and Treatment Planning, and weakest on Core Counseling Attributes and Ethics. Generalist models outperformed medical models on answer accuracy, although medical models performed better on several reasoning metrics. |
| Perlis et al., 2023; Evaluate GPT-4 antidepressant ranking in prescribing vignettes | Vignette; simulated, no real clinical setting; patients with depression in antidepressant treatment vignettes (n=10); Depression | Standardized antidepressant treatment vignettes were entered into the model one at a time. The model was asked to rank antidepressants from best option to contraindicated. Each vignette was regenerated multiple times to examine consistency. The intervention involved treatment ranking from brief prescribing scenarios. | models: GPT-4 (ChatGPT Plus) prompting info: Structured ranking prompt evaluation date: April 12 to 14, 2023 | Expert clinician consensus rankings served as the reference standard; whether the optimal treatment was included among the best choices and whether poor or contraindicated treatments were incorrectly included | Across 50 vignette runs, the model selected the optimal treatment in 76.0% of cases, but also included contraindicated or poor options in 48.0%. Performance was perfect in seven vignettes, mixed in one, and failed entirely in two. Frequent hallucinated drug interactions and incorrect contraindication handling limited safety and reliability for independent prescribing. |
| Perlis et al., 2024 [67]; Test guideline-augmented GPT-4 for bipolar treatment selection | Vignette; simulated, no real clinical setting; patients with bipolar disorder in clinical vignettes (n=50, expanded evaluations n=1000); Bipolar depression | Clinical vignettes were entered into the system in two modes. In one mode, the model responded using only the vignette; in the other, treatment guidelines were added to the prompt. The model was asked to select the top next-step pharmacologic treatments. The intervention tested whether adding guideline text changed medication recommendations. | models: gpt-4-1106-preview (GPT-4-turbo) prompting info: Structured clinical prompting with and without guideline augmentation; temperature 0 evaluation date: Not explicitly reported | Expert consensus served as the reference standard, with community clinicians as an additional comparator; agreement with expert-defined optimal treatment, inclusion of contraindicated treatments, and comparison versus clinicians | The guideline-augmented model substantially outperformed both the base model and clinicians for bipolar treatment selection. Top-treatment accuracy was 50.8% versus 23.4% for the base model and 23.1% for clinicians; top-3 and top-5 inclusion of the correct treatment reached 84.4% and 94.9%. Contraindicated or poor treatments were still selected in 12.0% of evaluations. |
| Silva and Gomes, 2025 [68]; Evaluate retrieval-augmented LLMs for psychiatric medication support | Vignette; simulated, no real clinical setting; psychiatric inpatients in a multicenter EHR dataset (n=9923); Depression | For each clinical case, patient history, current medication, current situation, and clinician questions were combined. The system retrieved relevant medication-document passages and inserted them into the prompt together with the case information. The LLM then answered medication-related queries under different query formats. The intervention involved retrieval-augmented medication decision support. | models: gpt3.5; gpt4o; deepseek-r1; llama3; llama3.1; llama3.2; medllama2; nemotron-mini; phi4; starling; text-embedding-3-small for embeddings prompting info: Role-based prompting with injected context, patient record, current situation, and question, requesting direct answer, complete answer, advantages, and disadvantages evaluation date: Not explicitly reported | Author-defined expected replies for each case served as the reference standard; manual scoring of whether the model considered patient context correctly, chose the right medication, explained its answer adequately, produced high-quality response text, and respected the requested format | Across 40 medication-related psychiatric cases, the best overall system was gpt4o_chat with a mean score of 4.10/5.00, narrowly ahead of gpt4o_query at 4.07. Commercial models outperformed open models overall, mainly because open models struggled with output-format adherence. Query-style prompting generally outperformed chat, although gpt4o was a small exception. |
| Tan et al., 2025 [70]; Evaluate LLMs on psychotherapy case-response tasks | Vignette; simulated, no real clinical setting; psychotherapy case vignettes (n=3 cases: depression, anxiety, stress/burnout) with 12 therapy-related prompts per case; General mental health | Seven LLMs were given three psychotherapy case vignettes and asked to respond to 12 structured prompts for each case. The prompts covered case formulation, treatment planning, treatment implementation, session structure, first-session simulation, technique recommendations, and goal-setting. All models were tested using zero-shot prompting. Responses were collected, anonymized, and rated by clinical psychologists. | models: ChatGPT-4o; ChatGPT-4; Claude 3.5 Sonnet; Claude 3 Opus; Llama 3.1; Gemini 1.5 Pro; Microsoft Copilot prompting info: Zero-shot evaluation date: July 2024 | Human expert evaluation by 5 clinical psychologists; mean Likert ratings (1-5) across 12 therapy tasks, inter-rater reliability, nonparametric comparisons, and qualitative thematic analysis | Across 3 cases, 12 prompts, and 7 models, no LLM achieved consistently acceptable performance, with overall mean ratings around 2.74 to 2.88, below the threshold of 3.0. Performance was relatively better for session duration and goal-setting discussion, but weaker for case formulation, first-session simulation, and most treatment-planning tasks. Qualitative feedback noted strengths in structure and technique suggestions but weaknesses in clinical reasoning, individualization, safety, and integration across steps. |
| Thotapalli et al., 2025 [71]; Evaluate GPT-4 psychiatric emergency triage from vignettes | Vignette; simulated, no real clinical setting; patients with psychiatric emergencies in simulated triage vignettes (n=21); Psychiatric emergencies | Psychiatric emergency vignettes were entered into the models. The models were asked to assign triage decisions about risk level, urgency, and hospital admission. Each vignette was run multiple times. The intervention involved simulated mental health triage from emergency scenarios. | models: GPT-4o; GPT-4o Mini; GPT-4 Legacy prompting info: Structured vignette-based triage questions evaluation date: August to September 2024 | Clinician triage decisions by two nurse practitioners served as the reference standard; agreement with clinicians on admission decision, risk level, and urgency, plus analysis of false positives and false negatives | GPT-4 models showed substantial agreement with clinicians for psychiatric emergency triage, with κ values of 0.76-0.78 across admission, risk, and urgency judgments. GPT-4o and GPT-4 Legacy outperformed GPT-4o Mini, and there were no false negatives, although four false positives suggested over-triage. Findings support use as decision support rather than replacement. |
| Zhang et al., 2025 [17]; Benchmark LLM competence across CBT knowledge and skills | Vignette; simulated, no real clinical setting; CBT benchmark datasets spanning knowledge, classification, and therapist-response tasks; General mental health | LLMs were evaluated on a benchmark covering three levels of CBT competence. The models answered CBT multiple-choice questions, classified cognitive distortions and core beliefs from patient-like text, and generated therapist responses to simulated patient statements. Zero-shot prompting was used for knowledge and classification tasks, and structured prompting was used for response generation. | models: GPT-4o; Mistral-v0.3-7B; Gemma-2-9B; Llama-3.1-8B; Llama-3.1-70B; Llama-3.1-405B prompting info: Zero-shot for Levels I and II; structured prompting for Level III evaluation date: Not reported | Human expert performance, ground-truth labels for classification tasks, reference therapist responses for CBT-DP, and pairwise expert comparison of LLM versus human responses; accuracy for Level I, precision/recall/F1 for Level II, and expert pairwise preference scoring for Level III | For CBT knowledge questions, performance was high, with Llama-3.1-405B achieving 95.0% accuracy and GPT-4o 94.1%, both exceeding human performance. Performance on cognitive understanding tasks was more modest, and for therapeutic response generation all LLMs underperformed human reference responses. Qualitative findings highlighted strengths in structured reasoning and guidance, but weaknesses in empathy, rapport, validation, flexibility, and patient-centeredness. |
| ***Treatment Support*** | | | | | |
| Acevedo et al., 2026 [72]; Compare ChatGPT with human CBT therapists in simulated sessions | Vignette; simulated, no real clinical setting; mental health therapy interactions with transcript evaluations by professionals (n=75 raters); CBT-related mental health problems | The LLM was given the same clinical scenario and prompts as a human CBT therapist. It generated a full text-based therapy session transcript in response to the scenario. The conversation was designed to resemble a complete CBT session rather than a single answer. The intervention was simulated delivery of a text-based CBT session. | models: ChatGPT-3.5 prompting info: Identical priming dialogue and scenario as used for the human therapist evaluation date: October 2023 to September 2024 | An experienced human CBT therapist served as the comparator; mean CTRS scores across domains, proportion of respondents giving top ratings, and p-values for between-group differences | Human therapists outperformed ChatGPT-3.5 across most CBT competence domains, with significant differences for feedback elicitation, collaboration, pacing, guided discovery, focus on key cognitions, CBT technique use, homework assignment, and several other domains. ChatGPT was described as more generic, rigid, and impersonal, and more likely to ask insufficient questions and fail to seek feedback. |
| Alanzi et al., 2024. Aim: Evaluate ChatGPT as treatment support for adults with anxiety disorders. | Prospective: real-world outpatient setting; public hospital care plus home LLM use; adults with anxiety disorders (n=399), outpatient clinical population; anxiety disorders | Participants were asked to use the LLM as a therapist over a 4-week period. They were instructed to have regular conversations about anxiety, stress, and daily experiences, and could use different therapeutic styles depending on preference. The intervention involved repeated, self-directed text conversations over time. | models: ChatGPT (GPT-3.5, free version); prompting info: role-based prompting with therapist persona and guided user prompts; evaluation date: not explicitly reported | No formal comparator or control group; outcomes: survey-based ratings of comfort, helpfulness, empathy, trustworthiness, and effectiveness, analyzed with t-tests and ANOVA | Among 399 patients with anxiety disorders, 91.2% reported that ChatGPT-3.5 understood their anxiety accurately. Ratings were generally favorable, with high or moderate comfort in 66.4%, high or moderate helpfulness in 70.2%, and moderate or high empathy in 59.0%; mean scores were 3.51 for trustworthiness, 3.53 for effectiveness, and 3.54 for clarity/coherence. The most common concerns were privacy (67.2%) and ethics (64.9%), while 28.7% reported concern about lack of human connection. Younger patients, females, and urban users rated ChatGPT more positively than older, male, and rural users, with significant subgroup differences across multiple comparisons (p<0.05). |
| Haber et al., 2025 [74]; Evaluate AI-generated feedback for suicide-prevention role-play training | Prospective; simulated, no real clinical setting; general population adults (~n=48) with simulated conversational data (n=54 conversations); Suicidality | Participants engaged in real-time text conversations with a simulated suicidal individual. After the conversation, a second AI persona generated structured feedback and a quantitative performance score. The simulation imposed specific constraints on how suicidality was disclosed during the exchange. The intervention combined role-play practice with automated feedback. | models: GPT-4o (OpenAI) on the PMFM AI platform prompting info: Custom prompt architecture without fine-tuning or retrieval augmentation evaluation date: June 6, 2024 | Mean ratings from three trained human psychology raters served as the comparator reference; correlation between GenAI scores and human ratings, ICC agreement, mean score bias, and qualitative thematic analysis | AI-generated QPR feedback correlated strongly with human ratings (r=0.721, p<0.001), with ICC 0.827, compared with human inter-rater ICC of 0.897. The AI tended to score performances 1.2 points higher than humans on average but showed no significant gender bias. |
| Hodson & Williamson, 2024 [75]; Evaluate LLM performance on CBT-style cognitive distortion tasks | Vignette; simulated, no real clinical setting; patients with cognitive distortions related to depression/anxiety, using therapist-generated CBT examples (n=20 tasks); Cognitive distortions | The models were given short CBT-style tasks across three stages: generating examples, identifying cognitive biases, and reframing unhelpful thoughts. Each task was presented separately. The models completed the full set of tasks across all stages. The intervention focused on core CBT-style text generation and interpretation. | models: ChatGPT-4; Google Bard prompting info: Structured task-based prompts for CBT exercises evaluation date: June 2023 | Human CBT therapists served as the gold-standard evaluators; proportion of correctly completed tasks, total score out of 60, and therapist judgments of correct bias identification and reframing quality | Both models performed moderately well across CBT tasks, with ChatGPT-4 scoring 44/60 (73.3%) and Bard 42/60 (70.0%). Performance was best for reframing thoughts, where Bard outperformed ChatGPT-4 (19/20 vs 16/20), while ChatGPT-4 performed better at identifying cognitive bias (15/20 vs 10/20). Both models performed similarly for generating initial vignette content (13/20 each). |
| Jain et al., 2024 [76]; Evaluate an AI-supported journaling system for mental well-being | Prospective; simulated, no real clinical setting; general population users (n=50) using AI journaling app entries; General mental well-being | Users wrote journal entries about thoughts, feelings, and daily experiences in a digital app. The AI responded in real time with reflective, supportive, and guidance-oriented messages. The system also used conversation memory and journal history to shape later replies. The intervention involved repeated AI-supported journaling over time. | models: LLM not explicitly specified; implemented using LangChain prompting info: Structured prompt templates with instruction-based prompting evaluation date: Not explicitly reported (conference 2024) | No direct comparator was used; Likert-scale responses, Pearson correlations, and percentage agreement on perceived benefits across counselling skills, behaviour changes, and mental health knowledge | The counseling system received high ratings across core therapeutic skills, behavior, and learning domains. Agreement was 82-90% across counseling skill items, with strong significant subscale correlations ranging from r=0.40 to 0.89. |
| Levkovich & Elyoseph, 2023b [53]; Compare LLM depression treatment recommendations with physicians | Vignette; simulated, no real clinical setting; patients with depression in simulated vignette scenarios (n=8); Depression | Depression vignettes were entered into the models. The models were asked to recommend treatments such as psychotherapy, medication, combined treatment, or watchful waiting. When medication was recommended, they were also asked to specify the drug type. The intervention was treatment recommendation from brief depression scenarios. | models: ChatGPT-3.5 and ChatGPT-4 (May 24, 2023 versions) prompting info: Vignette-based prompting with a structured treatment question evaluation date: June 2023 | Primary care physicians served as the comparator group; agreement with treatment guidelines, frequency of treatment types, and chi-square comparisons versus physicians | For depression vignettes, ChatGPT recommended psychotherapy far more often than physicians for both mild and severe depression, while physicians more often favored pharmacotherapy. No gender or socioeconomic bias was observed in ChatGPT outputs, whereas physician responses showed bias. |
| Levkovich et al., 2025 [58]; Compare LLMs with psychotherapists in training on behavioral activation tasks | Prospective; simulated training setting, no real clinical setting; adults with and without depression using clinical interview transcripts from DAIC-WOZ (n=189 sessions); Depression | Psychotherapists in training completed a behavioral activation questionnaire and case-scenario assessment before and after a teaching period. The LLMs completed the same pretest and posttest tasks, and at posttest they were also given the training materials as context. For case scenarios, prior model responses within the same case were preserved. The intervention was standardized psychotherapy knowledge and response generation under training and post-training conditions. | models: GPT-4; GPT-4o; Gemini Pro 1.5; Claude Opus; Llama-3 70B Instruct; Command R+ prompting info: Identical German system prompt defining an expert psychotherapy role, followed by multiple-choice case-scenario prompts with preserved within-case chat history and posttest training-material context evaluation date: Responses were generated between April and May 2024 | Psychotherapists in training served as the comparator group; multiple-choice knowledge score, expert-rated adherence, empathy, validation quality, anticipation of cognition, anticipation of emotion, counts of six validation strategies, and ICC interrater agreement | LLMs performed similarly to or better than psychotherapists in training on behavioral-activation knowledge and case-scenario responses. Open-ended ratings consistently favored LLMs, with significant advantages for cognition and emotion anticipation at pretest and for adherence and empathy at posttest. |
| Moëll, 2024; Compare GPT-4 and ChatGPT-3.5 on common psychological questions | Vignette; simulated, no real clinical setting; patients with mental health conditions using prompt-based LLM responses (n=18 prompts); General mental health | The models were prompted with 18 predefined psychological questions. Each model generated an independent written response to every question. The focus was on how the LLM answered common mental health queries. The intervention was direct question answering on psychological topics. | models: GPT-4 and ChatGPT (exact ChatGPT version not explicitly reported) prompting info: Standardized fixed prompt set of 18 questions with blinded human rating evaluation date: Not explicitly reported | GPT-4 was directly compared against ChatGPT-3.5, using ratings from a licensed clinical psychologist as the reference; mean evaluator rating scores on a 1-10 scale and statistical comparison of mean scores | GPT-4 outperformed ChatGPT-3.5 across all 18 psychological prompts, with mean scores of 8.29 versus 6.52, and was rated as more empathic, clinically relevant, and actionable. Both models performed less well on PTSD, pain management, and sleep-disorder prompts. |
| Napiwotzki et al., 2025 [77]; Evaluate LLM psychotherapy knowledge and response quality before and after training materials | Vignette; simulated, no real clinical setting; patients with psychiatric disorders in DSM-5-TR clinical case vignettes (n=106); Depression | Psychotherapists in training completed a behavioral activation questionnaire and case-scenario assessment before and after a teaching period. The LLMs completed the same pretest and posttest tasks, and at posttest they were also given the training materials as context. For case scenarios, prior model responses within the same case were preserved. The intervention was standardized psychotherapy knowledge and response generation under training and post-training conditions. | models: GPT-4; GPT-4o; Gemini Pro 1.5; Claude Opus; Llama-3 70B Instruct; Command R+ prompting info: Identical German system prompt defining an expert psychotherapy role, followed by multiple-choice case-scenario prompts with preserved within-case chat history and posttest training-material context evaluation date: Responses were generated between April and May 2024 | Psychotherapists in training served as the comparator group; multiple-choice knowledge score, expert-rated adherence, empathy, validation quality, anticipation of cognition, anticipation of emotion, counts of six validation strategies, and ICC interrater agreement | LLMs performed similarly to or better than psychotherapists in training on behavioral-activation knowledge and case-scenario responses. Multiple-choice scores were numerically higher for LLMs, and open-ended ratings consistently favored LLMs, especially for cognition/emotion anticipation, adherence, and empathy. |
| Shen et al., 2024 [79]; Evaluate LLM-generated CBT-style responses with and without knowledge augmentation | Retrospective; retrospective psychotherapy transcript dataset, no live clinical setting; patients with mental disorders in a psychotherapy conversation dataset (n=196 sessions, mixed real + simulated); CBT-related mental health problems | Models were asked to act as CBT therapists in both single-turn and short multi-turn conversations. They generated therapeutic responses to mental health scenarios either with or without access to a CBT knowledge base. The same conversation tasks were repeated across systems. The intervention was CBT-style conversational response generation. | models: ChatGPT-3.5-turbo; ERNIE-3.5; iFlytek Spark V3.0; ChatGLM-3 prompting info: Instruction-based prompting with a CBT therapist role, multi-turn context conditioning, and CBT knowledge-base augmentation evaluation date: Not explicitly reported (conference 2024) | A human CBT therapist dialogue dataset served as the reference standard; semantic similarity to human CBT dialogues, emotional appropriateness, dialogue structure and coherence, and inquiry behavior | LLMs showed feasible CBT-style dialogue generation, with stronger performance in multi-turn than single-turn settings and further gains when a knowledge base was added. ERNIE Bot had the strongest sustained multi-turn performance, while ChatGPT performed best in single-turn proactive questioning; responses were more positive than human therapists, suggesting overly positive bias. |
| ***Documentation and Monitoring*** | | | | | |
| Adhikary et al., 2024 [80]. Aim: Evaluate LLMs for structured summarization of counseling-session content. | Retrospective: retrospective dataset; no live clinical setting; therapy-seeking individuals, counseling session transcripts (n=191 sessions, 11,543 utterances); general mental health | Full counseling-session transcripts were entered into the LLM, and each session was processed as an individual case. The model was asked to generate separate summaries for predefined therapy components rather than one overall summary, producing structured aspect-based summaries for each conversation. | models: GPT-2, GPT-Neo, GPT-J, BART, T5, Flan-T5, Llama-2, and Mistral; prompting info: full counseling-session transcripts entered with instructions to generate separate summaries for predefined therapy components; evaluation date: not reported | Human-annotated reference summaries, with additional head-to-head comparison against other LLMs; outcomes: ROUGE-1, ROUGE-2, ROUGE-L, BERTScore, precision, recall, F1, and qualitative expert evaluation of affective attitude, burden, ethicality, coherence, opportunity cost, perceived effectiveness, and hallucination | Best performance was achieved by MentalLlama, MentalBART, and Mistral. MentalLlama showed the strongest quantitative summarization performance, with ROUGE-1 F1 of 30.86 versus 28.00 for MentalBART. Mistral scored highest qualitatively for affective attitude at 1.33 (SD 0.32) and effectiveness at 1.12 (SD 0.47). Hallucinations were uncommon, with none in about 75-77% of outputs, minimal hallucination in about 13-18%, and extensive hallucination in about 5-11%. |
| Cardamone et al., 2025 [81]; Classify mental health concepts from emergency EHR text | Retrospective; retrospective emergency department EHR dataset; patients with mental health conditions in a large-scale EHR dataset (~6.2M episodes); Mental health disorders | Unstructured EHR text was given to the LLM. The model was first asked to classify terms as mental versus physical health and then to assign them to more specific categories. This was done without training examples. The intervention involved structured concept labeling from EHR language. | models: GPT-4-turbo-2024-04-09 prompting info: Zero-shot prompting with temperature set to 0 evaluation date: Model snapshot dated April 9, 2024 | Consensus coding by two expert clinicians served as the human reference standard; agreement with clinicians using kappa, plus precision, recall, and F1-score | Performance was strongest for the binary mental-versus-physical distinction, with κ=0.77, precision 0.93, recall 0.93, and F1-score 0.93. Performance dropped for finer-grained categorization, with κ=0.62 and F1 0.65 across 42 mental health classes and κ=0.69 and F1 0.72 across 19 physical health classes. Accuracy varied by category, with particularly strong performance for substance use and weaker results for miscellaneous groupings. |
| Donnelly et al., 2025 [82]; Evaluate LLM scoring of suicide safety-plan documentation | Retrospective; real-world outpatient mental health clinic setting; patients at suicide risk with safety planning intervention texts (n=266 forms, 2210 responses); Suicide prevention | Written safety plans were entered into the LLM system. The models were asked to score the quality of each response section and provide reasoning for the score. Multiple prompting and scoring formats were tested for the same task. The intervention involved automated rating of safety-planning documentation. | models: GPT-4; LLaMA 3; o3-mini prompting info: Zero-shot prompting with a structured SPISA-based rubric evaluation date: Not explicitly reported (preprint posted March 2025) | Trained clinical coders using the SPISA scoring algorithm served as the reference standard; weighted F1-score, precision, recall, and confusion matrices | Across 266 safety plans and 2,210 responses, performance varied by section and by model. Best F1-scores were 79.12% for warning signs, 81.13% for internal coping, 76.56% for environment safety, and 91.99% for reasons for living; simplified 3-point scoring improved performance versus the original 4-point system. No single model dominated all tasks, although LLaMA 3 was the most consistent overall. |
| Gireesh et al., 2025; Evaluate LLM proofreading and extraction from addiction psychiatry notes | Retrospective; real-world clinical setting at a tertiary teaching hospital addiction treatment center with electronic health records; addiction psychiatry clinical notes (n=6500 notes; 3000 first contacts, 3000 follow-ups, 500 discharge summaries); Substance use / addiction psychiatry | Free-text addiction psychiatry clinical notes were entered into LLMs for two tasks. The models were asked to proofread and standardize the notes by correcting spelling, expanding abbreviations, and improving structure and clarity. They were also asked to extract structured substance-use information, including substances mentioned and time since last use. The same zero-shot instruction prompt was used across experiments. | models: Qwen-2.5 Instruct (1.5B, 3B, 7B); LLaMA-3.1-8B-Instruct; LLaMA-3.2 Instruct (1B, 3B); Phi-3.5-Mini-Instruct; GPT-4o (gpt-4o-2024-08-06) prompting info: Zero-shot instruction prompting with the same prompt across experiments; temperature 0.1 and top-p 0.95 evaluation date: Not reported | Human-annotated gold-standard proofread notes and structured information extraction labels; additional comparison with Microsoft Word Editor, jamspell, contextualSpellCheck, and CoEDIT; METEOR, BERTScore F1, sentence-level F1, token-level F1, readability indices, precision, recall, F1, substance-class detection accuracy, adequacy-of-information accuracy, exact match proportion for time since last use, and masked human preference with 95% CI and Fleiss kappa | In off-the-shelf proofreading, larger LLMs outperformed simpler non-LLM tools, although smaller LLMs were less consistent. Qwen-2.5-7B-Instruct and LLaMA-3.1-8B-Instruct showed the strongest proofreading performance among general LLMs, while GPT-4o performed strongly on information extraction, with mean F1 0.97 for substance-class detection, exact match 0.96 for time since last use, and mean accuracy 0.97 for adequacy of time-since-last-use information. Overall, off-the-shelf LLMs outperformed simpler NLP tools but remained suboptimal without further adaptation. |
| Janota & Janota, 2024; Evaluate LLM-generated psychiatric discharge summaries from structured data | Vignette; simulated, no real clinical setting; patients with psychiatric disorders in fictional clinical cases (n=2 scenarios); Psychiatric disorders | Structured bullet-point patient information was given to the LLM. The model was asked to turn this information into a full psychiatric discharge summary. Clinicians received the same case information and wrote their own summaries separately. The intervention involved automated report generation from structured clinical data. | models: ChatGPT-4.0 prompting info: Structured input with minimal training examples, resembling few-shot prompting evaluation date: Not explicitly reported (published 2025) | Human-written discharge summaries by physicians and psychotherapists served as the reference comparator; composite quality scores across criteria such as clarity, coherence, and completeness, plus time efficiency | AI-generated discharge summaries matched or exceeded human performance while being much faster. In the depression case, the best human score was 101 versus 97 for AI, but in the schizophrenia case the AI achieved the highest overall score at 103 compared with the best human score of 98. Completion time was consistently shorter for AI at 15 minutes versus 25-90 minutes for humans. |
| Matsumura et al., 2026 [84]; Evaluate LLM symptom scoring from psychiatric EHR language during clozapine treatment | Retrospective; real-world inpatient psychiatric setting at Osaka Medical and Pharmaceutical University Hospital; patients with suicidality in hypothetical clinical questions (n=30 scenarios); Treatment-resistant schizophrenia | Patient speech extracted from EHR notes was entered into the LLMs. The models were instructed to rate all BPRS symptom items for each record using structured output rules. Records were grouped into baseline and treatment phases to examine change over time. The intervention involved symptom scoring from routine clinical language during clozapine treatment. | models: gpt-oss-120b; GLM-4.5-Air; Qwen3-Next-80B-A3B-Instruct prompting info: Structured instruction-based prompting in Japanese with an expert assessor persona, BPRS scoring rules, item definitions, JSON output, and examples evaluation date: Source data covered 2015 to March 31, 2023; model inference date not explicitly reported | Human-rated PANSS baseline scores served as the main reference standard, with conventional NLP features used as supportive comparators; LLM-derived BPRS symptom ratings over time, change in linguistic features across treatment phases, repeated-measures ANOVA, linear mixed-effects models, post-hoc Dunnett-corrected comparisons, and correlations with baseline PANSS and conventional NLP measures | In 30 patients with treatment-resistant schizophrenia, LLM-derived BPRS scores captured significant symptom improvement during clozapine treatment. Significant decreases were seen for anxiety, conceptual disorganization, suspiciousness, unusual thought content, hallucinatory behavior, and depressive mood, while baseline LLM-derived symptom ratings correlated positively with human-rated PANSS measures for key psychotic and behavioral features. The analysis also found shifts in language use over time, including increased adjective use, reduced verb use later in treatment, and more positive emotional expression. |
| ***Patient Education and question-answering*** | | | | | |
| He et al., 2024 [86]; Evaluate LLM responses to autism-related patient questions | Retrospective; retrospective online consultation dataset in a real-world digital care setting; autistic patients (n=100) with real-world consultation questions (n=239); Autism spectrum disorder | Real-world autism-related consultation questions were collected from an online medical platform. Each question was entered into the LLMs to generate a written response. Physician responses to the same questions were retained as parallel answers. The intervention involved question answering for autism-related patient concerns. | models: ChatGPT-4; ERNIE Bot v2.2.3 prompting info: Direct naturalistic question input without few-shot prompting evaluation date: August 16-30, 2023 | Physician responses served as the gold-standard comparator; mean Likert ratings for relevance, correctness, usefulness, and empathy, proportion of preferred responses, proportion scoring at least 4, and statistical group comparisons | Physicians were preferred overall to both models, receiving 46.86% of evaluations compared with 34.87% for ChatGPT and 18.27% for ERNIE Bot. Physicians scored higher on relevance and usefulness, while ChatGPT scored highest on empathy and slightly exceeded physicians on correctness; ERNIE Bot performed worst across domains. Confidence intervals were reported for all rating domains, and the sample comprised 717 evaluations. |
| Li et al., 2026 [87]; Evaluate LLM responses to real-world mental health questions | Vignette; simulated, no real clinical setting; real patient questions from CounselChat (n=100 across 20 topics), expert evaluations of model responses (n=2000 QA evaluations), and adversarial dataset (n=120 questions; 1080 model responses); General mental health | LLMs were given real-world mental health questions from an online counseling forum and asked to generate open-ended, counseling-style responses. Each response was evaluated by multiple licensed mental health professionals using a structured rubric covering quality, empathy, specificity, medical advice, factual consistency, and toxicity. Clinician-authored adversarial questions were also used to probe weaknesses such as unsafe advice and lack of empathy. Each question was processed independently, and multiple responses were generated and rated per model. | models: GPT-4-0613; LLaMA-3.3-70B-Instruct; Gemini-1.5-Pro prompting info: Standard single-turn question-answer prompting evaluation date: Not reported | Human expert ratings from 100 licensed mental health professionals served as the reference standard, with comparison against online human therapist responses and LLM-as-judge evaluations; Likert-scale ratings across multiple domains, binary classification of medical advice, inter-rater reliability (Krippendorff’s alpha), failure mode detection, and accuracy and F1 for LLM-as-judge evaluation | Across 2000 expert evaluations, LLaMA-3.3 showed the best overall performance, with high scores for overall quality (4.29), empathy (4.22), and specificity (4.63). GPT-4 had lower overall quality (3.28) and frequent refusal-style safety disclaimers, while Gemini showed lower empathy despite similar factual consistency. Human therapist responses scored lower overall and showed higher toxicity than LLMs. Unauthorized medical advice appeared in 7-14% of LLM responses, inter-rater reliability was high, and LLM-as-judge evaluation aligned poorly with human experts. |
| Yilanli et al., 2024 [88]; Evaluate GPT-4 psychoeducation for psychosis-related questions | Vignette; simulated, no real clinical setting; patients with psychosis using question-based scenarios (n=20); Psychosis | Common psychosis-related questions were entered into the LLM in new sessions. The LLM generated written answers to each question. Some questions were repeated to examine consistency of responses. The intervention involved question answering for psychosis-related patient or caregiver concerns. | models: GPT-4 (ChatGPT) prompting info: Zero-shot question answering with single-turn prompts per question evaluation date: Not explicitly reported (preprint July 2024) | Human expert evaluation by a psychiatrist and psychologist served as the reference standard; mean rubric scores for accuracy, clarity, inclusivity, completeness, clinical utility, and overall performance | GPT-4 provided highly accurate, clear, and clinically useful psychoeducation for psychosis-related questions. Mean scores were 2.89/3 for accuracy, 2.93/3 for clarity, 0.93/1 for completeness, 4.35/5 for clinical utility, and 3.55/4 overall. Inclusivity was the weakest domain (2.30/3), and readability was moderate, suggesting some responses may be difficult for some users. |
| Senturk and Koparal, 2026 [89]; Compare LLM and psychiatrist responses to antidepressant questions | Vignette; simulated, no real clinical setting; patients with psychiatric disorders using clinical cases and medication documents (n=40 cases); Depression and anxiety | Ten common antidepressant questions were paired with short patient scenarios. The LLM generated one written response for each scenario-question pair. Psychiatrists answered the same set of questions separately. The intervention was brief-answer generation for common antidepressant information needs. | models: ChatGPT-4o prompting info: Standardized prompt including the scenario and question text, with one response per question evaluation date: Not explicitly reported; only ethics approval dated July 30, 2024 was provided | Twenty-six psychiatrists served as the comparator benchmark, with blinded psychiatrist rating used as the reference process; accuracy rated on a 1-6 scale, conciseness rated on a 1-4 scale, readability by Flesch-Kincaid Grade Level, and clarity by Writing Clarity Index | ChatGPT-4o performed similarly to psychiatrists for accuracy on antidepressant-information questions, with no significant difference. It was significantly more concise, whereas psychiatrists produced significantly clearer responses. Readability did not differ significantly. |
| ***Patient Engagement*** | | | | | |
| Barabas et al., 2025; Evaluate GPT3.5 in crisis-support in psychiatric role-play | Vignette; simulated psychiatric clinic interaction, no real patient care; patients with psychiatric disorders in simulated role-play cases (n=20 scenarios); Acute psychiatric crises | Participants role-played patients and had free-text conversations with the LLM about psychiatric crisis situations. The LLM responded as a psychiatric first responder and offered guidance during the interaction. Each participant completed the conversation from the patient role. | models: ChatGPT (GPT-3.5) prompting info: Role-based prompting instructing the model to act as a psychiatric first-responder evaluation date: July to September 2023 | No human gold-standard comparator; participant-rated 10-point Likert scales for overall experience, helpfulness, pleasantness, realism, and appropriateness, analyzed with unpaired t tests, Fisher exact tests, and Welch ANOVA with Games-Howell post hoc testing | Participants rated GPT-3.5 positively overall, with mean scores of 8.4/10 for overall experience, 8.25 for helpfulness, 8.1 for pleasantness, 7.0 for realism, and 8.4 for appropriateness. Ratings were lower for psychosis scenarios than for depression or adjustment disorder, and feedback suggested greater suitability for affective disorders than psychosis. |
| McBain et al., 2025B [91]; Examine LLM responses across suicide-risk query levels | Vignette; simulated, no real clinical setting; adults using suicide-related questions categorized by risk level (n=30 questions; 9,000 LLM outputs); Suicide risk | Suicide-related questions were first categorized by experts into risk levels. The same questions were then repeatedly entered into several LLMs. Researchers examined whether the LLMs answered directly or shifted to refusal, redirection, or crisis support. The intervention involved repeated probing of LLM responses to suicide-related queries. | models: GPT-4o mini; Claude 3.5 Sonnet; Gemini 1.5 Pro prompting info: Standardized single-turn queries using 30 predefined questions at temperature 0.7 evaluation date: October 2024 | Expert clinician risk categorization served as the reference standard; probability of direct response, analyzed as adjusted odds ratios from logistic regression for alignment of responses to low-risk versus high-risk queries | Across 30 suicide-related questions and 9,000 LLM responses, ChatGPT and Claude directly answered very-low-risk questions 100% of the time, whereas Gemini did so only 25% of the time; all three gave 0% direct responses to very-high-risk questions. The models did not clearly distinguish intermediate-risk categories. Compared with ChatGPT, Claude was more likely to give a direct response, whereas Gemini was much less likely. |
| Scholich et al., 2025 [92]; Compare LLM and therapist text-based mental health support | Vignette; simulated, no real clinical setting; patients with depression in fictional text-based mental health scenarios (n=10); General mental health | Researchers created two fictional first-person mental health texting scenarios. Popular LLMs were prompted with these scenarios, while therapists responded to the same scenarios one message at a time as if texting a real client. The full LLM conversations were then shown back to therapists for review. The intervention was simulated text-based mental health support across LLM and therapist responders. | models: GPT-4; Gemini 1.0 Pro; Claude 3 Sonnet; Replika; Pi; Character.ai; Chai prompting info: Two scripted fictional scenarios were delivered as five to six sequential user messages evaluation date: May 1, 2024 for LLM versions at data collection | Licensed human therapists served as the comparator; frequency of therapeutic intervention types using MULTI coding, overall word count, therapist rankings, and qualitative themes on strengths, limitations, safety, and ethics | Therapists used fewer words than LLMs but were more likely to evoke elaboration, whereas LLMs used more suggestions, reassurance, psychoeducation, and affirmation. LLMs showed some useful therapeutic elements, but therapists judged them as too directive, generic, and potentially unsafe in crisis situations. Performance varied by scenario, with ChatGPT rated highest in one scenario but with no significant difference in the other. |
