# Supplementary material for: Generative Large Language Models in Mental Health Care Settings: Systematic Review and Meta-Analysis
Source: JMIR AI. 2026 Aug 31;5:e87730. doi: 10.2196/87730 (PMC13528826; doi:10.2196/87730)
Supplement: Supplementary file 4 [file ai-v5-e87730-s004.docx]

# Supplementary Material 2. PRISMA-S Checklist

| **PRISMA-S checklist item** | **Item** | **Location where item is reported / statement** |
| --- | --- | --- |
| **Information Sources and Method**s | | |
| **Database name**: Name each individual database searched, stating the platform for each where relevant. | 1 | Methods, Search Strategy: PubMed, Embase, ACM Digital Library, IEEE Xplore, and Google Scholar. Consider revising manuscript text to specify platforms exactly where relevant. |
| **Multi-database searching**: If databases were searched simultaneously on a single platform, state this. | 2 | Not undertaken. Databases were searched individually rather than through a combined multi-database platform. |
| **Study registries**: Name any study registries searched. | 3 | No study registries were searched for this review. |
| **Online resources and browsing**: Describe any additional websites, search engines, or online resources used, and how they were searched or browsed. | 4 | Methods, Search Strategy: Google Scholar was searched, and results were screened in order of relevance until no further potentially eligible studies were identified. |
| **Citation searching**: State whether backward and/or forward citation searching was conducted and how. | 5 | Methods, Search Strategy: We manually searched the reference lists of relevant systematic reviews and included articles to identify additional records. Forward citation searching was not undertaken. |
| **Contacts**: State whether authors, experts, or organisations were contacted to identify additional studies. | 6 | No authors, experts, or organisations were contacted to identify additional studies. |
| **Other methods**: Describe any other methods used to identify records. | 7 | No other search methods were used beyond database searching, Google Scholar searching, and manual reference list searching. |
| **Search Strategies** | | |
| **Full search strategies**: Include the full line-by-line search strategies for each database and information source, exactly as run. | 8 | Supplementary Material 4. |
| **Limits and restrictions**: Specify and justify any limits or restrictions, such as date, language, or publication status. | 9 | Methods, Search Strategy and Selection Criteria: Searches covered November 2022 to March 2026. No language restrictions were applied at the search stage or exclusion criteria, although all included studies were published in English. Peer-reviewed articles, preprints, conference papers, and abstracts were eligible if they met the criteria. |
| **Search filters**: State whether published search filters were used and cite them, or state that no published filters were used. | 10 | No published search filters were used. Search terms were developed by combining concepts related to large language models and mental health. |
| **Prior work**: Indicate whether search strategies were adapted from prior reviews, protocols, or other work. | 11 | Search strategies were developed for this review based on the review question and eligibility criteria. They were not directly adapted from a previously published review. |
| **Search Process** | | |
| **Dates of searches**: For each source, report the date last searched. | 12 | Methods, Search Strategy: The search was first conducted on 11 January 2025, updated on 26 June 2025, and updated again on 16 March 2026. |
| **Peer review**: Describe whether the search strategy was peer reviewed, and if so, how; if not, explicitly state this. | 13 | The search strategy was not formally peer reviewed using a structured process such as PRESS. |
| **Record Management** | | |
| **Total records**: Document the total number of records retrieved from each source and overall. | 14 | Figure 1. PRISMA flow diagram of study selection: Total records (4,088): Pubmed (1,915), ACM (397), IEEE (399), Embase (857), Google Scholar (520) |
| **Deduplication**: Describe the processes and tools used to remove duplicate records. | 15 | Methods, Study Selection: All records were imported into Covidence for deduplication and screening. Results, Study Selection: 404 duplicate records were removed. |
| **Record management**: Describe how records were managed throughout the search and screening process. | 16 | Methods, Study Selection: All records were imported into Covidence for deduplication and screening. Title/abstract and full-text screening were conducted independently by two reviewers. |
